# Effectiveness assessment of maternal and neonatal health video clips in knowledge transfer using neuromarketing tools: A randomized crossover trial

**DOI:** 10.1371/journal.pone.0215561

**Published:** 2019-05-08

**Authors:** Laura Prieto-Pinto, María Fernanda Lara-Díaz, Nathaly Garzón-Orjuela, Dayanne Herrera, Carol Páez-Canro, Jorge Humberto Reyes, Lina González-Gordon, Viviana Jiménez-Murcia, Javier Eslava-Schmalbach

**Affiliations:** 1 Hospital Universitario Nacional de Colombia, Facultad de Medicina, Universidad Nacional de Colombia, Bogotá, Colombia; 2 Departament of Human Communication, Facultad de Medicina, Universidad Nacional de Colombia, Bogotá, Colombia; 3 Technology Development Center, Sociedad Colombiana de Anestesiología y Reanimación–S.C.A.R.E., Bogotá, Colombia; 4 Department of Psychology, Facultad de Ciencias Humanas, Universidad Nacional de Colombia, Bogotá, Colombia; Monash University, AUSTRALIA

## Abstract

Audiovisual educational material has been used effectively as a knowledge translation strategy in patient education. Given the need to impact maternal mortality rates, 12 video clips related to maternal and neonatal health information were designed based on the results of a previous systematic review (SR). The content was formulated based on clinical practice guideline recommendations and validated following a formal consensus methodology. This study evaluated the effectiveness of knowledge transfer from the 12 video clips in terms of attention, emotional response, and recall by using neuroscience tools. In a randomized cross-over trial, 155 subjects (pregnant women, non-pregnant women, and men) received random sequences of 13 video clips, including a control video clip. Participants’ attention levels were evaluated through eye tracking, their emotional reactions were monitored by electrodermal activity and pupillary diameter, and their recall was tested via a questionnaire. An analysis was performed to evaluate differences in the groups and between the video clips and the control clip using variance analysis models that considered period, sequence, and carry-over effects. Results revealed that fixation length was greater in women than in men, while the greatest emotional effects occurred in men. All three groups had good recall results, without any significant differences between them. Although the sequencing did influence attentional processes, no carry-over effect was demonstrated. However, a differential effect was noted among video clips in all three outcomes, that is, when adjusted for group, level of education, and having had children. The control clip generated less attention, emotional reaction, and recall than the experimental video clips. The video clips about maternal and neonatal health were shown to be effective in the transference and comprehension of information. Therefore, cognitive neuroscience techniques are useful in evaluating knowledge translation strategies through audiovisual formats.

## Introduction

Improving maternal health and reducing maternal mortality are priorities in public health. At the global level, the importance of intensifying the efforts of national action plans in this regard is reinforced through the prioritization of the health of women and children [1]. In Colombia, the maternal mortality rate in 2017 was 51.4 deaths per 100,000 live births (LV). Maternal mortality is higher among the poorest population groups and four times higher in the departments where the highest Multidimensional Poverty Index rates have been reported (Vichada and Chocó) [2].

In order to identify the causes of high maternal mortality, a systematic analysis was developed by the WHO [3], which revealed that for the period 2003–2009, 73% of all maternal deaths were the direct result of obstetric problems, with hemorrhage and in particular postpartum hemorrhage being the most frequent direct cause. It is evident, therefore, that the highest proportion of maternal morbidity and mortality occurs during childbirth, strategies must be formulated to tackle these issues. Although many complications of pregnancy are not preventable, early recognition of warning signs and timely consultation prior to childbirth and in postpartum stages can have a positive effect on maternal outcomes, with a lower proportion of complications and better health results [4]. Strategies focused on reducing the number of maternal deaths should be directed not only at health professionals and institutions, but also at the pregnant woman, her family and friends, and the community at large. Everyone involved should receive clear and specific information to know how to recognize warning signs and act on them in a timely manner.

The existing gap between knowledge generated through research and its application has been recognized as one of the most critical public health challenges we face today, and knowledge transfer (KT) can address this gap [5–7]. Consequences of the knowledge-action gap included the suboptimal use of effective treatments, poor health outcomes, and health inequalities, which have a negative impact on quality of life, productivity, and resource use and distribution [8]. KT strategies can be used to evaluate the implementation of knowledge and identify methods to reduce the gap between the generation of evidence and its application in clinical practice [9].

The use of reminders and video clips ensures that scientific evidence will be accessible to decision-makers and health providers [10]. A systematic review (SR) that assessed the effectiveness of intervention strategies determining factors in clinical practice focused on improving outcomes included 15 studies in its quantitative analysis. The review reported an OR of 1.56 (95% CI 1.27–1.93, p < 0.001) in favor of targeted interventions [11]. Similarly, the effectiveness of knowledge strategies in public health has been demonstrated [11,12]. However, few strategies facilitate the empowerment of patients regarding their health care, specifically those for pregnant women and children.

Cognition is the ability to recognize and process information by using knowledge to modify preferences; in problem-solving it is related to reasoning, learning and drawing conclusions [10, 11]. Memory is made up of associations that represent events, people or places. However, memory is selective, and in general, what people remember is what they have deemed to be important or interesting. Each type of information is represented by specific neuronal patterns, and the repetition of a pattern strengthens the recall of an event in the process known as long-term potentiation [12]. Thus, for example, the repetition of a message, as occurs in the case of advertising campaigns, should stimulate recall of the information pertaining to the advertised product.

Although social scientists commonly use research tools such as interviews or self-declared questionnaires, these are insufficient to gather all the necessary data [13], because the responses to these tools reflect introspective reflections about the emotions in relation to an advertising stimulus. Reactions to subtle stimuli may be impossible to quantify using traditional methods. An alternative is to evaluate autonomic responses concentrating on emotional reactions that are not distorted by higher cognitive processes [14]. Other techniques have been developed using cognitive neuroscience to observe the processing and making of decisions in real time in which aspects of perception, attention, emotion, and memory come into play [15,16]. Neuromarketing has emerged as an area in which the balance of internal and external preferences that govern emotional experiences is being studied and understood through behavioral observation and physiological monitoring [17]. This allows the relationship between emotional and rational behaviors to be analyzed, particularly in relation to marketing and persuasion [18,19].

A method widely used in neuromarketing is eye tracking, a technique that allows the detection and recording of eye movements. During the execution of a visual task, the relationship between eye movements and cognitive processes such as language and image processing, memory processes, social cognition, and decision making can be examined [20,21]. Eye tracking is used to understand how a person perceives, explores, searches, and remembers what has been visualized. This provides a window into the cognitive processes involved with the process of visualization [22]. Calvert et al. evaluated the use of the eye tracking technique to predict the effectiveness of advertising campaigns that had been previously rated as good or bad. The researchers reported significant differences in eye tracking for each type of campaign in terms of fixation on the number of quadrants per second. The finding suggest that commercials are more effective when attention is kept on fewer areas of the screen [23]. Oculometric data are useful in this research because they can be used to improve the selection of appropriate video clips based on their ability to be remembered and their emotional impact. Additionally, the measurement of neuropsychological reactions can be monitored based on the analysis of subtle changes in the galvanic skin response [19]. Therefore, the clear wording and appropriate understanding of the message that is received, as well as the activation of the neural mechanisms of attention and emotion, will enable the prediction of the retention ability and the effectiveness of the received stimulus.

Thus, the facilitation of the dissemination of health knowledge can also be based on the results obtained from cognitive neuroscience strategies applied to marketing, or neuromarketing, as it is known in the fields of economics and advertising [17]. These strategies could contribute to the knowledge and empowerment of pregnant women and their families, along with better care in the prenatal, childbirth, and postpartum processes. It would, therefore, improve the health outcomes for both the mother and the newborn child. The objective of this study was to evaluate the effectiveness of 12 video clips in the KT of information about maternal and neonatal health by measuring levels of attention, emotional response, and recall through using neuromarketing tools.

## Materials and methods

The protocol for this trial and supporting CONSORT checklist are available as supporting information; see S1 Checklist and S1 File.

### Ethics statement

The study protocol was approved by the Research Ethics Committee of the Sociedad Colombiana de Anestesiología y Reanimación (S.C.A.R.E.) on July 7^th^, 2015 (No. CE201503). The trial was registered before participants were recruited on the international trial register (ClinicalTrials.gov: Identifier NCT03330860). The authors confirm that all ongoing and related trials for this intervention are registered.

### Study design and participants

**Fig 1:**
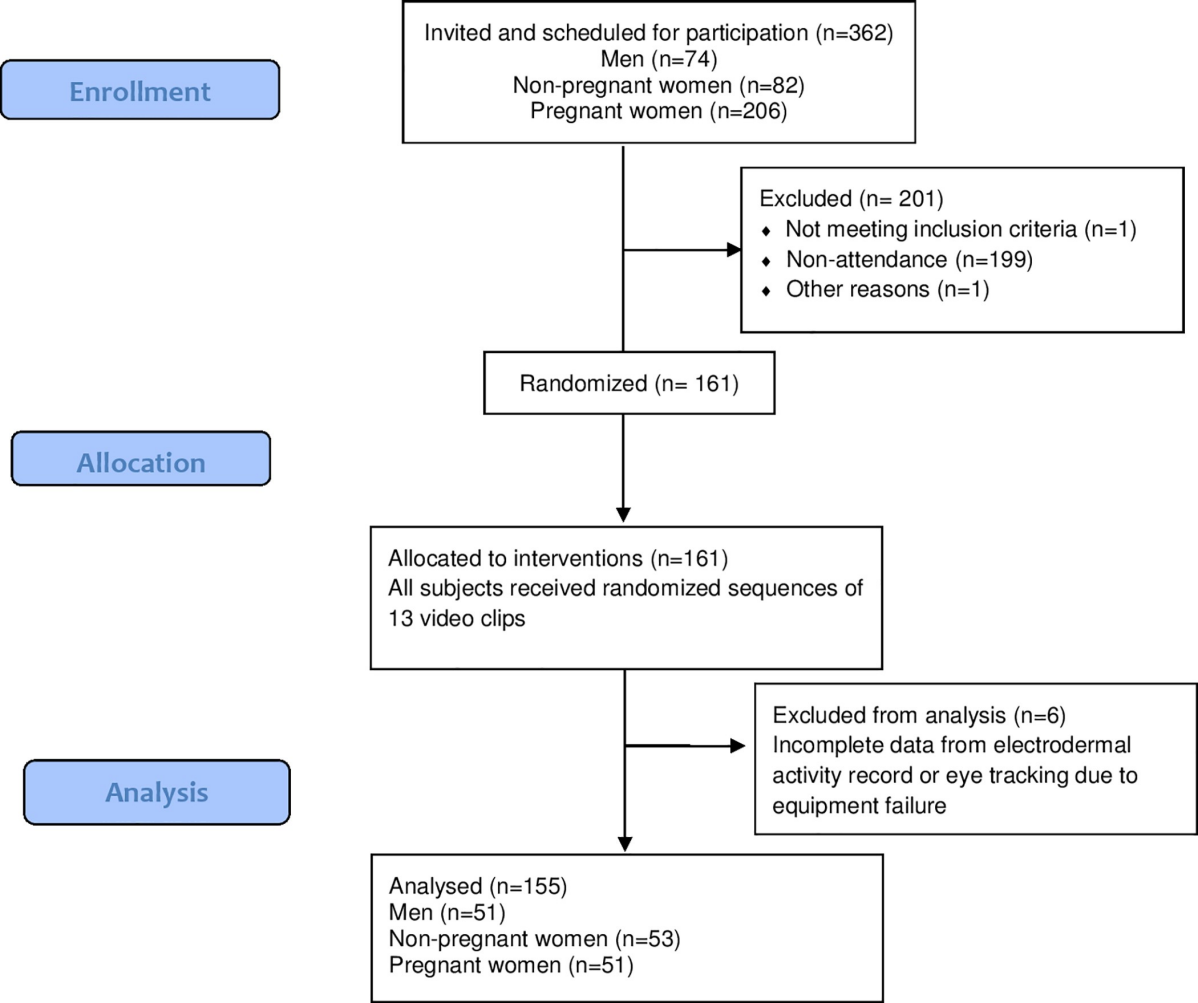
CONSORT flow diagram of subject recruitment, allocation and analysis.

A randomized, open crossover trial was conducted, where each participant included in the study received randomly-generated sequences of 13 video-clips (one of them as a control). The randomization method was based on the algorithm designed by the eye tracker (Tobii TX300), which uses the Latin squares design to create the sequences, constructing (x) number of presentation sequences, according to (n) number of video clips selected to be randomized, and defining video clip 13 (the control) as the basis for randomization. Through this method, the allocation concealment was also guaranteed, since neither the patient nor the researcher knew the presentation sequence of the videos. Due to the nature of the interventions and the outcome measurement method, blinding of subjects or researchers was not possible. The sample was obtained through intentional, non-probabilistic sampling; participants included were pregnant women, non-pregnant women, and men over 18 years of age. Subjects were excluded if they displayed health problems related to pregnancy or some clinically significant visual or auditory disorder. The CONSORT flow diagram of subject recruitment, allocation and analysis is shown in Fig 1.

#### Intervention

Each subject was exposed to 13 video clips containing information regarding maternal and neonatal health. These consisted of:

Intervention videos: 12 video clips about prenatal control (video clips 1 to 4), labor and delivery care (video clips 5 to 8), and postpartum and newborn care (video clips 9 to 12) were made. Each video aimed at providing important information during each of these stages. These video clips were made with mixed 2D and 3D elements. Every video was about 45 seconds long, and their format and content were prepared based on the best available evidence, obtained from a SR (Phase I) [24]) and clinical practice guidelines, which was validated by clinical experts (Phases II and III) (S2 File). The decision regarding the duration of each video was based on the fact that segmentation allows learners to engage with small pieces of new information, which facilitates control over the flow of new information; therefore, each video included only key information on a single topic. Similar interventions using short videos have proven to produce better engagement than using longer videos [25,26]. The areas of interest (AOIs) of each video clip were defined as the most relevant areas that best represented the key concepts and on which the participants were expected to fix their attention more (S2 File).

Control video: video clip in 2D, with information about prenatal control presented in a conventional format (narration, static images and text on the screen). It was obtained from the link: https://www.youtube.com/watch?v=VghQlqtX71U. A section of the video was used for the purposes of this study.

After each video there was an eight second rest period (wash out period), during which a neutral sequence was presented with the intention of calibrating the response of the individual in a neutral state and thus, be able to control the carry-over effect. The participation of the subjects was voluntary, and the measurements were carried out by trained personnel, after obtaining written informed consent. The content of the videos was not explained either before or during the test. Participants were recruited between May and June 2017 through phone calls, online calls and social networks. The sessions were held in the Laboratory of Cognitive Neuroscience and Communication of the National University of Colombia, from June 23 to July 21, 2017, with an approximate duration of 30 minutes per participant.

#### Outcomes

Attention and emotional reaction outcomes were measured through techniques used in neuromarketing: eye movements and pupillary diameter through the eye-tracking method, and electrodermal activity measurement employing a galvanometer.

Attention: the eye-tracking method was used to evaluate the subject´s attention to the visual stimulus of the video clips, using the Tobii Tx300 Eye Tracker equipment. Eye movements were recorded during each video presentation. The following variables were measured, which registered how and when was the subject’s focus on the AOIs: 1) time to first fixation (TFF) in seconds on an AOI (indicative of the efficiency of the arrangement of visual elements [27]), and 2) duration of each of the fixations made on an AOI (has been shown to be related to the area’s information, content or complexity [28]). These measurements indicate the total duration of the fixation on each AOI.

The screen-time of each AOI and the duration of subject fixation on each AOI was recorded in seconds. A proportion between the subject fixation time and the screen-time of each AOI was then calculated to make the results comparable despite the differences in AOI screen-time. Subsequently, to obtain a final measurement per subject, an average of the data was calculated, obtaining the proportion of fixation time over AOI screen-time for each video. To obtain the time to the first fixation on an AOI, the time elapsed from the beginning of each AOI to the exact time of fixation on the area was measured. Then for each subject, the average time to first fixation per video clip was calculated.

Emotional reaction: the monitoring of the autonomous activity to evaluate the subjects´ emotional state or reaction was carried out by recording electrodermal activity. This phenomenon is related with the sweat glands´ response to a stimulus, which alters the skin´s electrical properties [29]. The electrodes were placed on the distal phalanges of the third and fourth fingers of the non-dominant hand, with SE-35 sensors connected to a T-601 preamplifier (J & J Enterprises Seattle, WA). Physiolab USE3 Software was used for the analysis of results (J & J Engineering, 2004). The analysis was performed by calculating the amplitude of the electro-dermal response (μS) to a stimulus [29]. Two values were required to calculate the amplitude for each AOI: the conductance recorded at the lowest point during the latency period (baseline), and the conductance peak. Thus, for this study, we analyzed the data from a phasic perspective. The baseline was the beginning of each video where the title of the topic to be addressed was presented and no other images were displayed. This analysis was conducted for each subject for each AOI per video-clip, and measurements where a positive amplitude was obtained were included in the data: that is, those in which the skin conductance measurement was greater than that of the baseline, since positive amplitudes are those associated with an emotional impact.

In addition to the recording of the electrodermal activity, the emotional reaction was also evaluated by measuring the pupillary diameter (in mm) per millisecond with the visual tracking equipment (the eye tracker), since the reaction of the pupil is a psychophysiological indicator of cognitive and emotional processing [30]. Although the variations of the pupil are usually related to the cognitive load, they do not allow researchers to determine the intensity or emotional valence [31]. However, some studies have inferred emotional impact through this technique [32]. Recent research has confirmed that the pupil dilates in response to emotionally arousing materials [33,34]. In consumer science, various studies have reported increases in pupil size in response to stimuli and experiences that evoke strong interest and emotions in consumers [35–37].

Additionally, pupil dilation is associated with the activity of the locus coeruleus [38] which is a key region of emotional arousal, as it reflects activity of the SNS induced by the presentation of emotional stimuli and indicates intense emotional arousal toward both pleasant and unpleasant stimuli and experiences [39]. Pupillary responses appear to indicate emotional arousal, increased mental activity in decision making and in response to rewards [39]. An average measurement of the pupillary diameter of each eye was taken for all the video clips, and a final average of both eyes.

Recall evaluation: The assessment of the recall component was carried out by having participants complete a knowledge test in the form of a questionnaire after each video clip had been watched. The questionnaires, each with two or four questions, were prepared by the researchers based on the information presented in each video clip and on the AOIs. As the number of questions per questionnaire differed between video clips, once the total of correct answers per questionnaire had been established, the proportion of correct answers was calculated with respect to the total number of questions (S3 File).

### Missing data

In total, 6 subjects were excluded from the analysis as a result of incomplete recording of the electrodermal activity or ocular tracking due to equipment failure. In total there were 19 sets of missing data (6 for the fixation time in AOIs, 6 for the time of first fixation, and 7 for the electrodermal activity), corresponding to missing measurements for some videos owing to technical failures in the measurement of the electrodermal activity and eye tracking. These data were imputed using the last observation carried forward method [40,41].

### Sample size

For the calculation of the sample size, the statistical power analysis program G* Power 3 [42] was used for a one-way analysis of variance (ANOVA) and fixed effects, using the F statistic. The expected sample size (f) was estimated based on the classification described by Cohen [43], which defines values of 0.1, 0.25 and 0.4 as small, medium and large effects, respectively. The selection of parameters used for the recall results were an average effect size f = 0.3; power (1-β) = 0.90 and alpha (α) = 0.05 for the three groups. The calculation yielded a result of a required total sample of 144 subjects. As there was to be no monitoring of the participants and the possible number of losses (mainly due to failures in recording), a minimum of 150 subjects was defined for the total sample.

### Statistical analysis

A descriptive analysis of the data was carried out, with the qualitative variables presented in proportions and the quantitative variables by measures of central tendency and dispersion depending on their distribution (Shapiro-Wilks test). An overall analysis was made for the levels of attention, emotional response and recall among groups (non-pregnant women, pregnant women and men), by level of education and having had children, using the Kruskal-Wallis test or Wilcoxon sum rank test, depending on the number of levels of each factor. As a result of the multiple comparisons between attention, emotional reaction and recall, the level of significance was adjusted to a p<0.0025, using the Bonferroni correction method for multiple comparisons [44,45].

Because this is a crossover trial, one of the main drawbacks is the carry-over effect: this effect can be controlled or prevented by introducing a wash-out period between interventions, which must be long enough to eliminate the effects of a process [46]. In this case, a wash-out time of 8 seconds was used for all subjects between the 13 video clips. An analysis was carried out for the levels of attention, emotional reaction and recall, adjusted by group (men, non-pregnant women, and pregnant women), level of education, and children, using the statistical package pk, in particular pkshape and pkcross, of the software STATA 13.1. This allows the analysis of crossover experiments by analysis of variance models (ANOVA) taking into account period, sequence and carry-over effects. Because the sample data do not come from a normally distributed population, it was necessary to carry out the transformation of the variables, with the square root method for the attention variable, logarithmic for the emotional reaction variable, and the cube power for the recall variable.

## Results

The final sample consisted of 161 subjects, with an average age of 27 years, and with a similar distribution among the three groups. The proportion of subjects by level of education was similar among groups, with a higher number of participants at the high school and undergraduate levels (77%) (Table 1).

**Table 1:** Sociodemographic characteristics of the participants.

Variable	Man (N = 54)	Non-pregnant woman (N = 56)	Pregnant woman (N = 51)	Total sample (N = 161)
Age Median (Q1-Q3)^1^	24 (21–30)	27.5 (22–47)	30 (23–35)	27 (22–35)
Level of education	n (%)	n (%)	n (%)	n (%)
None	0	2 (3.5)	0	2 (1.24)
Elementary	1 (1.8)	4 (7.1)	6 (11.8)	11 (6.8)
High School	25 (46.3)	25 (44.6)	26 (51)	76 (47.2)
Undergraduate	18 (33.3)	15 (26.8)	15 (29.4)	48 (29.8)
Postgraduate	10 (18.52)	10 (17.9)	4 (7.8)	24 (14.9)
Children	n (%)	n (%)	n (%)	n (%)
Yes	6 (11.1)	21 (37.5)	33 (64.7)	60 (37.3)
No	48 (88.9)	35 (62.5)	18 (35.3)	101 (62.7)

^1^Q1-Q3: first quartile–third quartile

**Fig 2:**
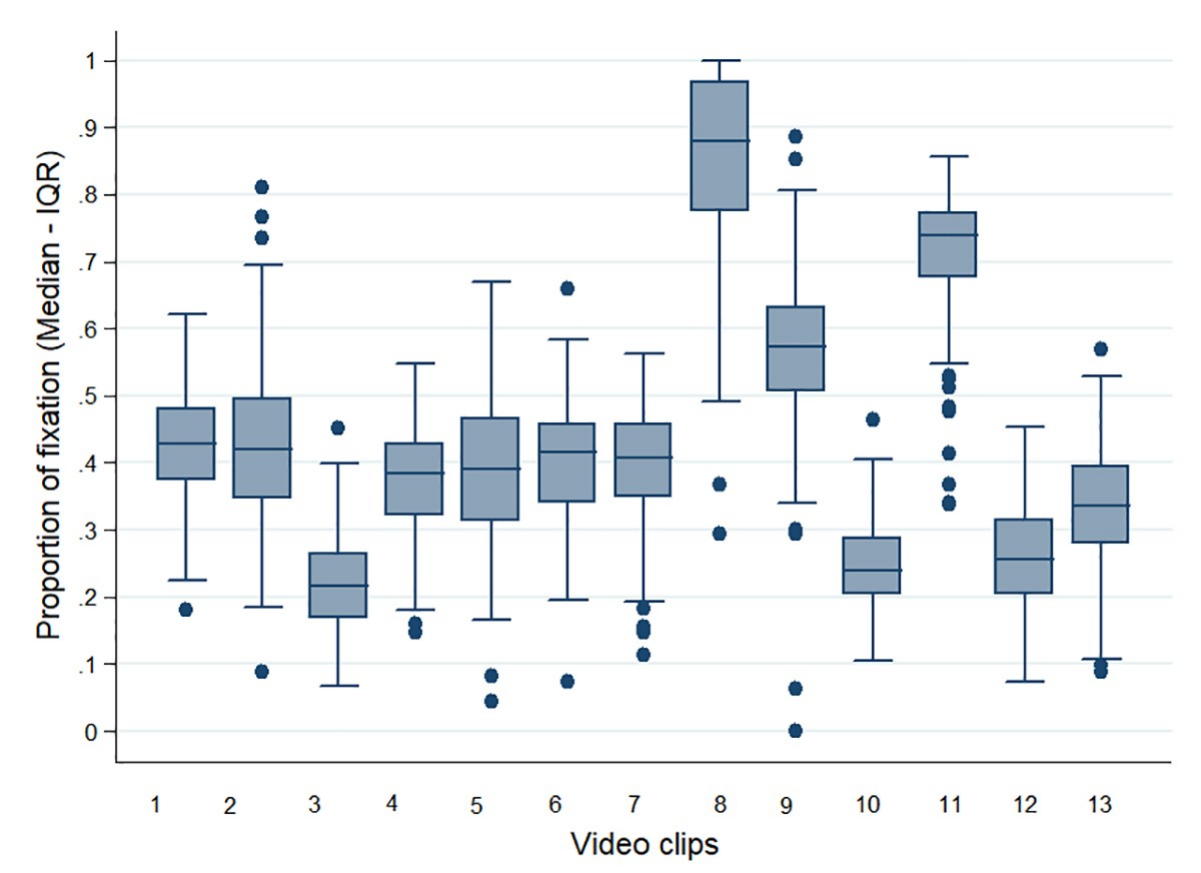
Proportion of fixation in areas of interest (Median—(IQR)).

**Fig 3:**
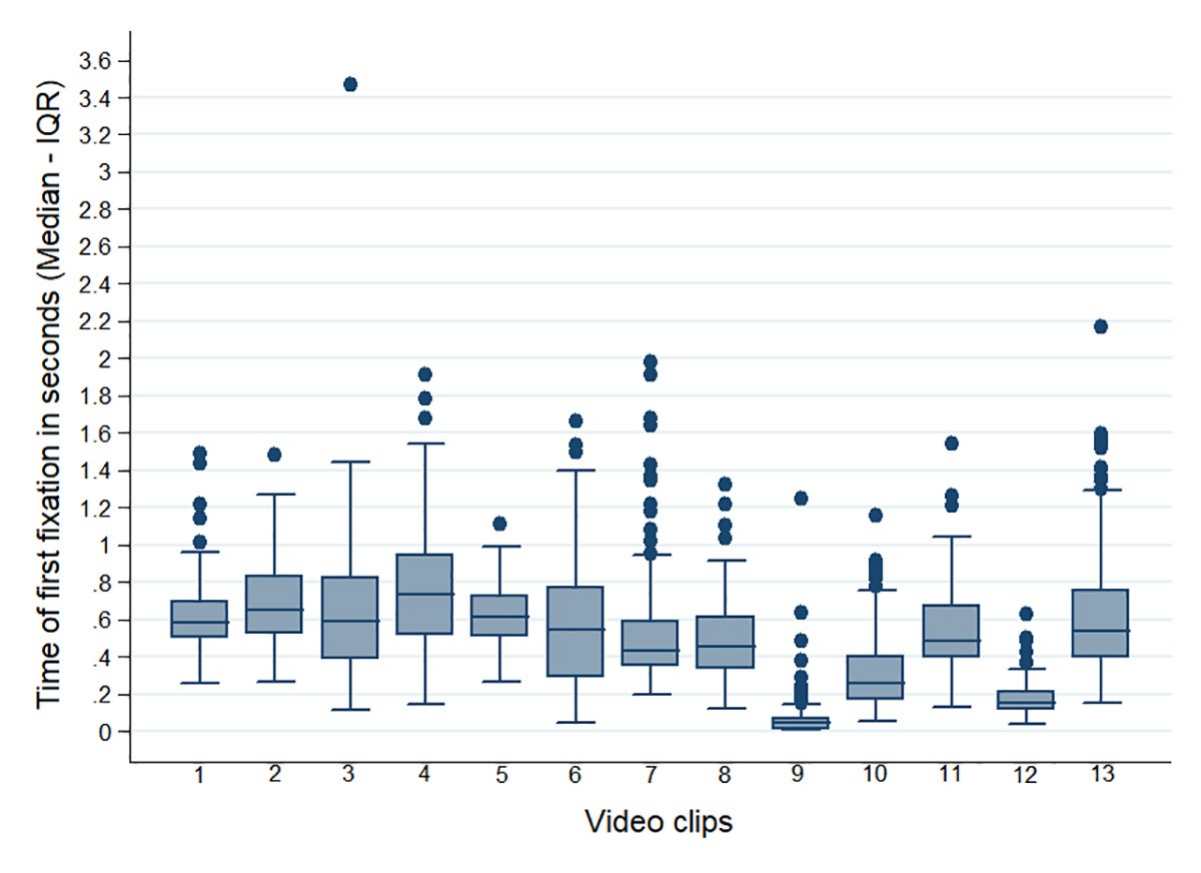
Time to first fixation (in seconds) in areas of interest (Median—(IQR)).

Attention: for all the videos, the proportion was higher in the group of pregnant women compared to the other two groups. Significant differences were observed among the groups for video clip 11 (p<0.0025) (Table 2). Fig 2 shows there was a longer fixation time in the AOIs of clips 8, 9 and 11, with proportions greater than 0.5. On the other hand, clips 3, 10 and 12 were reported as having the lowest result. In relation to the time to the first fixation, a greater homogeneity of the data between video clips was identified, with less than one minute elapsed before the first fixation in all videos, the times of clips 9 and 12 being the lowest and the time of clip 4 the highest, as shown in Fig 3. When comparing the proportion of fixation with respect to the total time of the AOIs for each video clip with the control video, significant differences were noted for all the comparisons (Table 2); however, for video clips 3, 10 and 12, this favored the control video.

**Table 2:** Time and proportion of fixation in areas of interest per groups of men, non-pregnant women and pregnant women (attention).

Video clips	Proportion of fixation in areas of interest (Median—(IQR^1^))				Time to first fixation (in seconds) in areas of interest (Median—(IQR^1^))			
	Man (n = 51)	Non-pregnant woman (n = 53)	Pregnant woman (n = 51)	Total n = 160	Man (n = 51)	Non-pregnant woman (n = 53)	Pregnant woman (n = 51)	Total n = 160
V-1	0.42 (0.36–0.50)	0.42 (0.36–0.46)	0.43 (0.39–0.47)	0.42 (0.37–0.47) **	0.62 (0.53–0.74)	0.58 (0.50–0.69)	0.55 (0.46–0.65)	0.65 (0.51–0.83) **
V-2	0.41 (0.34–0.49)	0.41 (0.32–0.49)	0.42 (0.37–0.49)	0.41 (0.34–0.49) **	0.71 (0.55–0.85)	0.66 (0.57–0.85)	0.61 (0.5–0.75)	0.59 (0.38–0.82)
V-3	0.21 (0.16–0.26)	0.21 (0.16–0.25)	0.23 (0.18–0.26)	0.21 (0.16–0.26) **	0.59 (0.41–0.82)	0.64 (0.51–0.94)	0.44 (0.33–0.75)	0.73 (0.51–0.94) **
V-4	0.36 (0.31–0.40)	0.39 (0.29–0.45)	0.39 (0.32–0.43)	0.38 (0.32–0.42) **	0.81 (0.54–0.96)	0.67 (0.47–0.85)	0.73 (0.49–0.94)	0.61 (0.51–0.72)
V-5	0.37 (0.29–0.44)	0.36 (0.30–0.45)	0.41 (0.34–0.50)	0.39 (0.31–0.46) **	0.59 (0.46–0.67)	0.61 (0.52–0.72)	0.64 (0.54–0.75)	0.54 (0.29–0.77)
V-6	0.42 (0.34–0.44)	0.40 (0.34–0.45)	0.41 (0.31–0.46)	0.41 (0.33–0.45) **	0.53 (0.26–0.71)	0.51 (0.23–0.77)	0.56 (0.39–0.77)	0.43 (0.35–0.59) **
V-7	0.38 (0.32–0.44)	0.40 (0.34–0.44)	0.45 (0.39–0.48)	0.40 (0.34–0.45) **	0.45 (0.35–0.70)	0.46 (0.39–0.58)	0.38 (0.32–0.57)	0.45 (0.33–0.61) **
V-8	0.88 (0.77–0.96)	0.86 (0.76–0.89)	0.88 (0.84–0.97)	0.88 (0.77–0.96) **	0.47 (0.32–0.60)	0.45 (0.35–0.67)	0.43 (0.32–0.60)	0.04 (0.01–0.06) **
V-9	0.56 (0.46–0.61)	0.57 (0.51–0.62)	0.58 (0.52–0.63)	0.57 (0.50–0.63) **	0.03 (0.01–0.06)	0.04 (0.008–0.06)	0.04 (0.01–0.08)	0.25 (0.16–0.40) **
V-10	0.23 * (0.19–0.26)	0.22 * (0.19–0.26)	0.25 * (0.22–0.31)	0.23 (0.20–0.28) **	0.30 (0.15–0.51)	0.25 (0.18–0.39)	0.23 (0.14–0.36)	0.48 (0.39–0.67)
V-11	0.72 (0.65–0.77)	0.71 (0.66–0.76)	0.75 (0.70–0.78)	0.73 (0.67–0.77) **	0.47 (0.39–0.64)	0.48 (0.39–0.62)	0.75 (0.70–0.78)	0.15 (0.11–0.21) **
V-12	0.23 (0.18–0.29)	0.26 (0.21–0.29)	0.26 (0.21–0.32)	0.25 (0.20–0.31) **	0.15 (0.11–0.20)	0.15 (0.12–0.21)	0.15 (0.10–0.22)	0.53 (0.39–0.75)
Control	0.31 (0.27–0.38)	0.33 (0.27–0.39)	0.35 (0.29–0.40)	0.33 (0.27–0.39)	0.51 (0.34–0.75)	0.56 (0.40–0.99)	0.49 (0.41–0.68)	0.58 (0.50–0.69)

^1^IQR: first quartile–third quartile

*Significant differences p<0.0025 between comparison of groups–non-parametric Kruskal-Wallis test.

**Significant differences p<0.0025 between comparison of each video clip with the control–Wilcoxon rank-sum test

Participants without education had the lowest fixation levels, and a similar trend was identified in non-pregnant women and men with elementary education. The differences between levels of education were observed mainly in video clips 2, 5, and 7; however, no significant differences were demonstrated. It was also observed that subjects without children had a longer fixation time in the AOIs, especially in the first six video clips, video clip 12 and the control video.

With respect to the time to first fixation, the data again showed better results in the group of pregnant women, that is, a shorter time to first fixation. The longest times were reported by men in six of the thirteen video clips; however, for the control video the longest time to the first fixation was that of non-pregnant women (Table 2). Regarding education levels, it was noted that the higher the level of education (undergraduate and postgraduate), the greater the time elapsed for the first fixation in the AOIs, especially in non-pregnant women. Significant differences were observed in video clip 3 (p = 0.002) and within the groups, there was no significant difference in any of the comparisons.

In the comparison between subjects with and without children, it was observed that for subjects without children, there was a shorter time to first fixation in the AOIs in each of the three groups, except for the first three video clips in the group of pregnant women. Video clip 9 showed the smallest difference between the two groups of having and not having children. The differences these two groups were observed mainly in video clips 11, 12 and 13, but were not statistically significant.

Table 3 shows the variability between subjects, owing to the presence of the sequence effect; this finding suggests that the order of presentation of the videos has an effect on the time of fixation. Likewise, there was intra-subject variability due to a period effect, but no carry-over effect was observed. Therefore, the time it takes the subjects to fix their attention on the AOIs differs between video clips, but this result does not depend on the effect of the previous video (Table 3, model 1). When including the factors of education level, group, and having had children, the model was adjusted appropriately to the data (F = 0.000) and showed 80% for the proportion of the time of fixation and 55% for the time of first fixation of the variability, as explained by the independent variables included (R2 = 0.79 and 0.55); that is, the time of fixation in the AOIs is significantly related to the levels of the three factors included (Table 3, model 2).

**Table 3:** Results of the outcome of attention in the analysis of variance models.

Proportion of fixation in areas of interest					Time to first fixation in areas of interest (seconds)			
Model 1								
Source of variability	gl^1^	Mean squares	Statistic test F	Prob > F	gl^1^	Mean squares	Statistic test F	Prob > F
Among subjects								
Effect of sequencing	27	0.05	1.99	0.0059	27	0.12	2.11	0.0030
Residuals	127	0.03	6.32	0.0000	127	0.06	2.42	0.0000
Intrasubject								
Carry-over effect	9	0.00	1.03	0.4172	9	0.04	1.51	0.1384
Period effect	32.35	2.70	667.70	0.0000	12	5.06	213.13	0.0000
Residuals	7.39	0.00			1830	0.02		
Model 2–Adjusted by group, education level, and children								
Model	37	1.7180	201.14	0.0000	37	1.7282	64.68	0.0000
Groups*	2	0.2721	31.86	0.0000	2	0.2867	10.73	0.0000
Education level	4	0.0427	5.00	0.0005	4	0.1094	4.09	0.0026
Children	1	0.4194	49.11	0.0000	1	0.6904	25.84	0.0000
Carry-over effect	9	0.0053	0.63	0.7705	9	0.0390	1.46	0.1563
Period effect	12	5.1580	603.86	0.0000	12	5.0606	189.39	0.0000
Residuals	1977	0.0085			1977	0.0267		

Analysis of variance (ANOVA) for crossover studies using the statistical package pkcross

^1^ gl = degrees of freedom

*Groups = Men, non-pregnant women and pregnant women

Emotional reaction: the analysis of electrodermal activity was carried out using the results of the positive amplitudes by AIOs of each video clip for each subject (emotional impact), which amounted to 14% of all the amplitude data, of which 31.6% corresponded to non-pregnant women, 37.3% to the group of men and 31% to the group of pregnant women. The electrodermal response amplitude used in the ANOVA model with pkcross corresponds to the mean of the transformed variable (since it did not show a normal distribution) of the positive amplitudes for each video. On the other hand, when making comparisons between groups and videos the variable that was not subjected to transformation (original data) was used, and therefore the results are presented as the median of the positive amplitudes for each video, together with the corresponding dispersion measure. The amplitude was greater in men, and significant differences were observed when comparing the results of this group with those of the group of pregnant women, in whom the amplitude of all video clips was less than 4 μS, suggesting a greater emotional impact in the men. Video clips 1, 6 and 11 produced a greater emotional response within the total sample, whereas video clip 5 and the control video were those that generated lower amplitudes; however, the differences between video clips are not significant (Table 4).

**Table 4:** Amplitude of electrodermal response and pupillary diameter by group of men, non-pregnant women and pregnant women (Emotional reaction).

Video-clip	Amplitude of electrodermal response in microsiemens (μS) (Median—(IQR^1^))				Pupillary diameter in mm (Median—(IQR^1^))		
	Man (n = 49) ^¶^	Non-pregnant woman (n = 52) ^¶^	Pregnant woman (n = 50) ^¶^	Total n = 151^¶^	Man (n = 51)	Non-pregnant woman (n = 53)	Pregnant woman (n = 51)
V-1	5.32 (2.11–12.59)	3.60 (1.89–12.04)	1.11 (0.76–2.87)	2.71 (0.94–6.77)	3.03 * (2.80–3.38)	3.15 * (2.88–3.45)	2.89 * (2.67–3.15)
V-2	3.04 (1.25–10.41)	9.24 (0.98–14.64)	1.58 (0.53–3.60)	2.15 (0.88–10.41)	3.09 * (2.84–3.41)	3.10 * (2.82–3.41)	2.86 * (2.63–3.13)
V-3	8.30 (3.94–11.73)	3.96 (0.74–14.06)	1.34 (0.52–2.94)	3.23 (0.85–8.92)	3.06 * (2.84–3.34)	3.05 * (2.81–3.34)	2.85 * (2.65–3.1)
V-4	2.92 (1.03–7.37)	2.28 (0.59–5.79)	1.70 (0.65–3.75)	2.47 (0.90–5.95)	3.23 * (2.99–3.55)	3.21 * (2.91–3.54)	2.95 * (2.70–3.22)
V-5	2.77 (1.50–8.31)	1.06 (0.20–4.01)	0.70 (0.51–2.71)	1.23 (0.52–3.71)	3.17 * (2.92–3.47)	3.15 * (2.86–3.44)	2.93 * (2.68–3.22)
V-6	3.77 (1.85–13.78)	2.71 (0.91–8.88)	0.76 (0.34–3.07)	2.63 (0.75–7.14)	3.14 * (2.91–3.44)	3.13 * (2.87–3.41)	2.88 * (2.68–3.15)
V-7	4.66 (1.45–10.03)	2.19 (0.74–3.72)	1.25 (0.57–2.99)	2.42 (0.83–5.37)	3.05 * (2.84–3.31)	3.06 * (2.8–3.37)	2.85 * (2.64–3.09)
V-8	3.69 (1.58–7.11)	0.90 (0.53–3.12)	1.42 (0.77–4.01)	2.44 (0.76–4.21)	3.09 * (2.86–3.38)	3.08 * (2.83–3.38)	2.88 * (2.67–3.14)
V-9	2.00 (1.70–6.49)	3.05 (1.64–9.36)	1.16 (0.75–3.01)	2.32 (0.98–5.77)	3.21 * (2.95–3.56)	3.24 * (2.94–3.59)	3.02 * (2.75–3.31)
V-10	2.83 (0.68–8.57)	3.12 (1.21–7.70)	0.71 (0.15–1.91)	1.92 (0.48–4.72)	3.17 * (2.94–3.46)	3.19 * (2.89–3.48)	2.90 * (2.7–3.19)
V-11	6.91 (2.03–12.14)	3.11 (0.58–14.30)	1.41 (0.69–3.34)	2.66 (0.98–11.40)	3.14 * (2.91–3.42)	3.17 * (2.85–3.48)	2.88 * (2.66–3.15)
V-12	3.29 (1.32–9.27)	2.21 (0.72–6.39)	1.51 (0.77–2.91)	2.20 (0.82–5.53)	3.18 * (2.95–3.48)	3.18 * (2.9–3.5)	2.92 * (2.72–3.2)
Control	3.28 (0.99–8.60)	1.44 (0.20–2.52)	1.06 (0.40–2.54)	1.46 (0.39–3.89)	2.94 * (2.74–3.19)	2.96 * (2.70–3.2)	2.73 * (2.57–2.96)

^1^IQR: first quartile–third quartile

^¶^ A lower sample is shown, as a result of subjects who did not produce a positive amplitude, i.e. those in which the measurement of conductance of the skin was less than that of the baseline.

*Significant differences p<0.0025 in the comparison of groups–non-parametric Kruskal-Wallis test.

The group of pregnant women showed the lowest amplitudes, but there were no obvious differences between levels of education. On the contrary, among men and non-pregnant women a greater emotional impact was observed in those with highest levels of education (undergraduate and postgraduate), but significant differences were only noted in video clip 10 (p = 0.001). In the comparisons between having and not having children, it was observed that in subjects who had no children there was a greater emotional impact among all three groups, although this finding was more evident in men and non-pregnant women; the differences between having and not having children for non-pregnant women were significant only in video clip 8 (p = 0.002).

For the majority of the videos, the pupillary diameter was highest in men, and significant differences were found for all the videos when comparing the groups (Table 4). The group of pregnant women displayed the lowest diameter for all the videos and thus was the group with the lowest emotional reaction. The smallest diameter recorded for each of the groups was for the control video (Table 4).

The result of the electrodermal activity in the analysis of variance did not show variability between subjects or intra-subjects; the effect of sequence was not proven, nor were the period and carry-over effects, so the emotional impact measured via electrodermal activity is not dependent on the differences between videos or on the effect of a video previously seen (Table 5, model 1). When including the factors of level of education, group, and having children, the model was adjusted in an appropriate manner to the data (F = 0.000). However, only 18% of the variability in the electrodermal activity is explained by the independent variables included (R2 = 0.178). The results of the model showed that the emotional reaction is significantly related to the levels of the three factors included (Table 5-model 2).

**Table 5:** Results of the emotional reaction outcome and recall in the analysis of variance models.

Amplitude of electrodermal response in microsiemens (μS)					Recall (proportion of correct responses)			
Model 1								
Source of variability	gl^1^	Mean squares	Statistic test F	Prob > F	gl^1^	Mean squares	Statistic test F	Prob > F
Between subjects								
Sequence effect	27	2.52	0.35	0.9989	27	0.18	1.08	0.3680
Residuals	123	7.25	4.89	0.0000	127	0.17	3.01	0.0000
Intrasubject								
Carry-over effect	9	1.71	1.15	0.3220	9	0.04	0.79	0.6241
Period effect	12	1.51	1.02	0.4324	12	0.79	14.27	0.0000
Residuals	1830	0.02			1830	0.06		
Model 2 –Adjusted for group, education level and children								
Model	37	9.5246	4.37	0.0000	37	0.5326	8.97	0.0000
Groups *	2	27.6107	12.67	0.0000	2	0.1095	1.85	0.1583
Education level	4	15.2786	7.01	0.0000	4	1.8745	31.58	0.0000
Children	1	42.4479	19.47	0.0000	1	0.4321	7.28	0.0070
Carry-over effect	9	1.5308	0.70	0.7072	9	0.4313	0.73	0.6848
Period effect	12	2.1323	0.98	0.4680	12	0.7882	13.28	0.0000
Residuals	745	2.1797			1977	0.0593		

Analysis of variance (ANOVA) for crossover studies using the statistical package pkcross

1 gl = degrees of freedom

*Groups = Men, non-pregnant women, and pregnant women

Recall: there were no statistically significant differences among the groups, with all the subjects in the sample answering correctly to most of the questions asked. While there was a good recall of the information in all the videos, the best-recalled video clips were those from 1 to 3 and 11, and the lowest recall was for the control video. In the comparison between each video-clip and the control, significant differences were identified in all the comparisons (p <0.001). Regarding the level of education, the results showed that the higher the level of education (undergraduate and graduate), the greater the information recall. The video clips 5, 8 and the control video produced the lowest scores among those with the lowest levels of education (none and elementary) and the control video produced the lowest scores in the rest of the levels.

In the variance analysis for the recall outcome, there was no variability between subjects, implying that there was no sequence effect, but there was intra-subject variability from a period effect. However, a carry-over effect was not evident, so therefore memory differs between video clips, but this response does not depend on the effect of the previous video (Table 5, model 1). When including the factors of level of education, group and having children, the model was adjusted in an appropriate manner to the data (F = 0.000); only about 15% of the variability in recall is explained by the independent variables (R2 = 0.143). The results of the model showed that recall is significantly related to the level of education and having children or not, but there were no differences between groups.

## Discussion

This study is one of the first to implement a neuromarketing strategy to evaluate an audiovisual KT tool in maternal and neonatal health. The study was done as a crossover trial, which also included within its development the implementation of research in epidemiology, cognitive neuroscience, and other disciplines. This study identifies methods to reduce the gap between formulating evidence and applying it in order to benefit patients [6,10,47]. It has become vital to design educational strategies that guarantee an effective learning process. Educational materials presented in an audiovisual format has already been used effectively in the education of patients [24,48,49].

Research in neuroscience aimed at the perceptions and reactions to commercial ads has heretofore focused mainly on evaluating the effectiveness of advertising campaigns in the field of neuromarketing [19,50,51] or predicting the effectiveness of public health campaigns. The objective is to optimize the messages and experiences for consumers by improving the effective transmission of those messages as well as modifying their beliefs, attitudes, preferences, and behaviors [16]. Examples of this include the anti-smoking campaigns [52].

Since a carry-over effect was not observed in any outcome the wash-out period implemented in this study was effective in eliminating the influence of a video clip on the one following it. It was also observed that the sequence effect, the order in which the videos were presented, did exert influence on the attentional processes, both in the time of fixation in the areas of interest and in the time to the first visual fixation. This is relevant because the study by Geiger et al., which evaluated participants’ attention to televisual clips, showed that subjects develop expectations about the next visual stimulus in a sequence based on the information of previously presented stimuli [53]. However, in this study, the order of presentation of the videos did not generally influence the recall of the video clip information or the emotional reaction to it. However, in the models adjusted by group, a distinct effect was observed between video clips, in the level of education and having children or not, for all the evaluated outcomes.

Between groups, there was a longer fixation time for the pregnant women, followed by the non-pregnant women this would seem to indicate that women tend to have greater attentional focus than men. Despite these differences, Video Clips 8, 9, and 11 had the longest fixation time across all three groups. The time to the first visual fixation followed a similar pattern and was quickest in the group of pregnant women as well. This would indicate that those who scan the screen more widely take a longer time to identify the AOIs. In this sense, the video clips were effective in attracting the attention of pregnant women in the AOIs, which represent the segments of special relevance. Similar findings were reported by Cárdenas et al., who found that visual attention was more stable in women than in men [54]. Other studies have shown, based on eye tracking techniques, that facial expression recognition is higher in women as well [55]. Gender differences in attentional processes are also supported in the selectivity model theory, which identifies men as selective processors and women as exhaustive processors [56,57]. In short, while in men message processing is done through getting a general scheme of the message, in women it produces a detailed wording of the content of the message, [58].

Subjects with lower educational levels had shorter visual fixation times. In some studies, it has been observed that individuals with a higher level of education support their visual tracking strategies with executive functions that favor concentrating one’s attention on the key elements of a scene [17,59,60]. Similar results have been described regarding the comprehension of visual stimuli based on levels of expertise. Thus, whereas experts tend to allocate attentional resources to relevant information, focusing on task-relevant areas and ignoring redundant images, novices show more variability on their areas of focus, and they have a longer duration of fixation on redundant areas [61]. However, in the group of pregnant women, this distinct pattern of fixation was not evident, suggesting that the closeness to the theme presented in the scenes favors the fixation on key elements and also diminishes the attention elsewhere.

The least-recalled video clip in all three groups was the control video clip, and the differences were significant when compared with each video clip. The control video clip differed from the others in several design aspects. It did not contain any 3D animation, the narration was presented in a foreign accent, and the video clip had background music, abundant text, and used static images. This finding reaffirms the results of the SR of the first phase and leads to the conclusion that the strategies implemented in the design of the experimental videos facilitated the attention and recall processes. Although no differences were found between the groups, as with the attention process the level of education indicated that the level of recall in the individuals with a lower educational level is lower; this could be related to the lower attention as observed in the times of fixation or to a lower short-term memory capacity in this group [62].

With regard to the familiarity effect, it was observed that the participants who did not have children were more focused on the video clips, displaying more time in fixation, a faster time of arrival to AOIs, and a greater percentage of recall. This is likely related to a greater influence of top-down attention, since these subjects had to reject other stimuli in the visual periphery that was equally interesting, that is, their interest guides their observations and facilitates the acquisition of knowledge [63,64].

The emotional reactions, measured both by recording electrodermal activity and by pupillometry, show that pregnant women have a lower emotional reaction to stimuli. In men, the stimuli had more of an emotional effect. Also, whereas pregnant women maintained a stable emotional state throughout their viewing of the video clips, the men and the non-pregnant women had more variable reactions. However, during the video clips that presented information on warning signs during pregnancy and postpartum care (Clips 4 and 12), pregnant women did react with higher pupillary diameter and amplitudes. Differences in terms of emotional reactivity between men and women have been previously reported; in this regard, women have been described as having a greater excitement in most emotions compared to men, which shows that when watching videos that induce an emotional response, men frequently show more intense emotional experiences, while women experience a greater emotional expressiveness. [65].

In general, the video clips that performed best in all the outcomes were Clips 8 and 9. This was possibly due to the fact that in Clip 8 (pain management) the narration of the information was supported by images that covered much of the screen, thus reducing the elements of distraction, and Clip 9 displayed highly emotional content, since it emphasized skin-to-skin contact with the newborn child and breastfeeding, with few elements of distraction.

Taking this study into account, it is clear that the design of educational audiovisual material for the health sector should use strategies that make the information easier to understand, such as minimizing distractions, presenting information of short duration, and using simple language with scenarios with which viewers can identify [66].

Some limitations of this study are: 1) the participants at the lowest level of education represented only 13% of the sample, limiting the generalizability of results for this subgroup; 2) there may have been possible conditioning effects for some participants because they knew in advance that they would be tested on the video-clip information (Hawthorne effect); and 3) the recall evaluation immediately followed the experimental presentation, and no follow-up evaluations were made.

The strengths of the research outcomes are that the participants had good retention and understanding of the information that was presented. Moreover, since the design and statistical analysis methods employed for this study are not commonly used in the evaluation of psychophysiological variables, even though frequently in this type of research, the measurements are often repeated on the same subject [50,67,68]. Therefore, the evaluation of variables that can influence the response, such as the order of presentation of stimuli and the carry-over effect, increase the internal validity of the research.

## Conclusion

The tools of cognitive neuroscience (neuromarketing) could become useful in the evaluation of attention, emotional response and memory in scenarios of educational strategies in health and public health interventions, which have the purpose of improving outcomes through the social acceptance of knowledge. Video clips of knowledge about maternal and neonatal health demonstrated greater effectiveness in the acquisition of knowledge, in terms of attention, emotional reaction and recall, compared with the control video. Audiovisual educational material must be clear, have short messages, and should not have distracting or redundant elements in order to reach a bigger proportion of the population.

## Supporting information

S1 Checklist Consort checklist. (DOCX)

S1 File Study protocol. (DOCX)

S2 File Phases of the evaluation study for the effectiveness of 12 video clips of knowledge transfer in maternal and neonatal health using applied neuroscience tools: A randomized crossover clinical trial. (DOCX)

S3 File Recall questionnaires of video clips 1 (prenatal control) and 4 (warning signs during pregnancy). (DOCX)
