# Supplementary material for: Effectiveness assessment of maternal and neonatal health video clips in knowledge transfer using neuromarketing tools: A randomized crossover trial
Source: PLoS One. 2019 May 8;14(5):e0215561. doi: 10.1371/journal.pone.0215561 (PMC6505891; doi:10.1371/journal.pone.0215561)
Supplement: S1 File — (DOCX) [file pone.0215561.s002.docx]

**S1 File. Study Protocol**

**SCIENTIFIC-TECHNICAL COMPONENT**

1. **Title of the project**

PILOT DESIGN AND STUDY TO EVALUATE OF THE EFFECTIVENESS OF 12 KNOWLEDGE CAPSULES IN MATERNAL AND NEONATAL HEALTH THROUGH NEUROMARKETING STRATEGIES.

1. **Principal investigator and co-investigators**
2. *Principal investigator*

- Javier Hernando Eslava Schmalbach

Time dedicated per week: 5 hours

Time spent on the project in months: 18

Duties: Coordination of design, methodology, project monitoring and publications.

1. *Co-investigators*

- Ana Carolina Amaya Arias

Time dedicated per week: 5 hours

Time spent on the project in months: 18

Duties: Support in the design of knowledge capsules, expert consensus, data analysis and publications.

- Sandra Viviana Jiménez Murcia

Time dedicated per week: 5 hours

Time spent on the project in months: 10

Duties: Support in the design of knowledge capsules, neuromarketing strategy, data analysis and publications.

- Juan Pablo Alzate Granados

Time dedicated per week: 10 hours

Time spent on the project in months: 18

Duties: Methodological support in RSL, expert consensus, neuromarketing strategy, data analysis and publications.

1. **Conformation of the research team**
2. **Investigation groups**

- Equity in Health Group, code COL0063855
- Quality, Safety and Health Education Group, code COL0041778
- Neurosciences Group, code COL000648

1. **Background and previous results of the research team tasked with the specific area of the project**

The Equity in Health Group, led by researcher Javier Hernando Eslava Schmalbach, aims to promote the development of research in equality in health. This group has worked on research projects related to patient safety, which address issues such as factors associated with long-term hospital stays, the development of bacterial resistance, and screening for adverse events, all related to obstetric care and the puerperium. Within the framework of these projects, the Equity in Health Group is actively involved in promoting the approach to equality in health in Clinical Practice Guidelines related to the detection, prevention and treatment of complications in pregnancy, childbirth or puerperium, to ensure that health inequalities do not widen, as well as in the equality component related to Clinical Practice Guidelines for the Syndromic Approach to the Diagnosis and Treatment of Patients with Sexually Transmitted Infections and Other Infections of the Genital Tract. The Equity in Health Group has focused on the measurement of inequalities in health outcomes, and has published articles related to the inequalities found in these results with respect to maternal mortality, neonatal mortality, mortality from cervical cancer, life expectancy for each department of Colombia, and the incidence of congenital syphilis, all of them related to contextual processes and care that affect the quality and result of the care provided, generally affected by a socioeconomic variable or affiliation to the Social Security in Health. Additionally, the Equity in Health Group actively participates as a speaker in seminars related to patient safety and is a member of the International Society for Equity in Health.

The Colombian Society of Anesthesiology and Resuscitation (S.C.A.R.E.) has been a national leader in the promotion of patient safety, both in anesthesiology and in other health professions, in particular directing its efforts towards the area of ​​obstetrics as this is one of the medical specialties with the greatest demand for safety strategies. In anesthesiology, it regularly holds the Colombian Congress of Anesthesiology and Resuscitation; it has scientific committees that deal, among other issues, with anesthesia safety; it has been in charge of creating a consensus for the minimum safety standards in anesthesiology applicable in Colombia and consensus related principally to safety in obstetric anesthesia, pediatric anesthesia and anesthesia for plastic surgery (Colombian Society of Anesthesiology and Resuscitation, 2006 and 2007); and it gains international recognition through the Colombian Journal of Anesthesiology, which is a reference for current knowledge in techniques, medicines, research and current standards in the field. The S.C.A.R.E. is also a leader in providing the ALSO and BLSO courses (Advanced Life Support in Obstetrics and Basic Life Support in Obstetrics, respectively) which, with periodic re-training in technical skills, improve safe practices in the mother-child environment of the obstetricians and anesthesiologists who perform obstetric anesthesia.

The Neurosciences Group at the National University of Colombia has worked on the investigation of the neurobiological aspects of neurodegeneration, movement disorders, human intellectual functions and neurodevelopment, with the purpose of facilitating the creation of new diagnostic, preventive and therapeutic strategies. It has investigated the identification of susceptibility genes and clinical characterization of the Colombian population for the most frequent neurological entities, as well as the study of clinical, genetic, epidemiological and pathological aspects of the most important and frequent neurodegenerative and neurodevelopmental diseases in the Colombian population.

The Laboratory of Neuroscience and Communication was created in 2014 as a meeting place for researchers from different areas whose interest is related to neurosciences and language. One of its lines of work is conducting neuromarketing studies.

Neuromarketing makes use of this information about the functions and mechanisms of the brain to help understand consumer behavior. Before the incorporation of different methodologies related to brain functioning, explanations about decisions made by the consumer were based on inferences; however, the tools offered by neuromarketing allow knowledge about the interactions between the human being and the market to have a solid scientific basis.

The introduction of tools, mostly cutting-edge technology, has rapidly transformed marketing in favor of the consumer. At present, neuromarketing allows different investigations to be carried out that go beyond the limits of traditional research, because they focus on the importance of creating value for the consumer and take into account the fundamental role that customer satisfaction plays in the generation of an innovative and competitive market.

In this regard, collaboration between companies and expert research groups in the field of neurosciences is essential. Neuromarketing not only favors consumer knowledge about the market, it also permits research studies to be carried out on the processing, cognition and emotions of human beings, in whom emotional and rational thought coexist. As a result, this combination of disciplines facilitates the advancement in knowledge of many key areas related to the new challenges that are imposed by the evolution of the market, an unavoidable aspect of modern-day society.

The interdisciplinary research group of the Laboratory of Cognitive Neuroscience and Communication seeks to promote an integral conception of Neuromarketing that promotes new theoretical and practical developments. The generation of biological models as a result of research in cognitive neuroscience that allow an understanding of the behavior of human beings in the context of consumption offers a more complete understanding of human behavior by incorporating points of view coming from different interdisciplinary approaches.

Since its creation, the laboratory has developed the diploma course "Fundamentals and Applications of Neuromarketing: from the senses to the decisions" which has been highly praised by its students. It has also developed different studies for entities such as Señal Colombia-Vive Lab, the Mayor's Office of Bogotá and the media agency of Unimedios at the National University.

1. **Research theme**

Maternal and perinatal health

Specifically in maternal and perinatal safety to reduce morbidity and mortality.

1. **Executive summary**

**Problem**

Improving maternal health and reducing maternal mortality are priorities in public health, highlighted as one of the eight Development Goals of the Millennium. Considering that the highest proportion of maternal deaths occur during the period of childbirth, it is necessary to generate strategies for the uptake and knowledge transfer (KT) in health focused on the effective communication of basic and relevant information in maternal and neonatal health to reach the general population. Knowledge transfer strategies, such as education, reminders and knowledge capsules, have been described as ways to ensure the use of scientific evidence by health professionals and decision makers to benefit patient health. However, little has been done regarding patients and the community, with strategies that facilitate the empowerment of patients and the community on the topic of health care, specifically regarding pregnant women and children, and how this could improve results related to maternal health. In light of the above, this research intends to answer the following questions: (1) What are the characteristics and relevant content in the design of strategies of social appropriation of knowledge in maternal and neonatal security aimed at patients and the community? (2) What is the effectiveness of 12 knowledge capsules in maternal and neonatal health to achieve the social appropriation of this knowledge in the target population?

**Aims**

**General:** Design and evaluate the effectiveness of 12 knowledge capsules in maternal and neonatal health through a pilot study to generate knowledge appropriation in the target population.

**Specific:**

- Determine the effectiveness of different strategies of social appropriation of knowledge through a systematic review of the literature.
- Based on scientific evidence and expert opinion, design 12 capsules of knowledge in maternal and neonatal health.
- Evaluate the effectiveness of the capsules in maternal and neonatal health in terms of social appropriation of knowledge, measured by attention, emotional response and recall through a pilot study, applying neuromarketing strategies.
- Produce a final version of the knowledge capsules in maternal and neonatal health with the design and content defined in the previous phases.

**Methodology**

The development of the project will be carried out in 6 phases:

1. Systematic review of the literature.
2. Preliminary definition of the contents of the knowledge capsules in maternal and neonatal health.
3. Formal consensus of experts under the modified Delphi method where the content and strategies to be used in them will be defined.
4. Design and development of health capsules in maternal and neonatal safety.
5. Evaluation of effectiveness of the knowledge capsules in maternal and neonatal health, applying a neuromarketing strategy, via an experimental cross-over study.
6. Final production of health knowledge capsules in maternal and neonatal safety.

**Analysis**

1. For the first phase of the project, corresponding to the RSL, a statistical combination of the results will be carried out independently, both for the randomized clinical trials and for cohort studies, through a meta-analysis, following an exploration of the presence of heterogeneity through the inconsistency test (I2) and the p-value of the chi-square test, and if there is variation in the definition of the evaluated outcomes. The statistical combination of the results will be made through meta-analysis of fixed effects, or in the case of heterogeneous data, a random effects model will be used. A sensitivity analysis will be carried out to study the influence of the risk of bias in the results of the meta-analysis.
2. For the neuromarketing strategy, corresponding to the final phase of the study, descriptive analysis of the data will be carried out. The normality of the variables will be evaluated by the Shapiro-Wilk test. The difference between the averages of the variables of emotional reaction and attention will be carried out by means of an analysis of variance (ANOVA) for dependent samples in case the variables are distributed normally, otherwise this difference will be analyzed by means of a Kruskal-Wallis test. For the analysis, statistics will be used for paired studies, taking into account that the subjects are part of the evaluation of all interventions.
3. For the results of the survey, in which the recall of the contents of the capsules will be evaluated, a proportional difference will be made between the groups with a good level of recall and those with a low level of recall for each category of educational level. The analysis of the collected data will be made using the statistical software STATA 12.0®.

**Expected results**

After the research is completed, there will be 12 health capsules for the preparation of childbirth, both for vaginal birth and for cesarean delivery, designed by means of neuromarketing strategies and the evaluation of their effectiveness on the social appropriation of the knowledge. It is hoped that the research, through this strategy of KT, will contribute to the development of educational material that provides knowledge of relevant and clear information for pregnant women and their families and will help to empower patients in maternal and neonatal health care. In the medium and long term it is hoped that the capsules will be used to be disseminated in a variety of mass media and in this way contribute to improve the knowledge and empowerment of the pregnant woman and her family, which will result in a better care in the stages of the childbirth and postpartum and in better outcomes in health for both the mother and the newborn child. Additionally, it is expected that one of the authors of this project will write their Master in Clinical Epidemiology thesis on this project, and that at least two articles will be published (one of scientific research and one of review) and a presentation will be made at an international event.

1. **Keywords**

Knowledge transfer, social appropriation of knowledge, patient safety, neuromarketing, maternal health, neonatal health

1. **Problem Statement**

Improving maternal health and reduce maternal mortality are priorities in public health, represented by objectives 4 and 5 (improving child health and maternal health, respectively) in the Development Goals for the Millennium, whose goal for 2015 is to reduce the Maternal Mortality Ratio (MMR) by 75% between 1990 and 2015 and to achieve universal coverage of specialized assistance at childbirth [1]. However, according to the United Nations report of 2008 [2], maternal mortality continued to be very high in developing countries. According to this report, in Latin America and the Caribbean maternal mortality rates were reduced by one third during the period from 1990 to 2005, but the estimated goal of 5.5% per year was not reached. Through the World Health Strategy for Women and Children in 2010 [3], this organization reiterates the importance of intensifying the efforts of the national action plans, by prioritizing the health of women and children.

In addition, although the complications of pregnancy, which result in obstetric emergencies are not preventable in many cases [4], early recognition of warning signs and timely consultation, both in stages prior to delivery and in its stages and care afterwards, will generate a positive impact, with a lower proportion of complications and better health outcomes. Therefore, the pregnant woman, as well as their families or companions, should receive clear and specific information to know and recognize these warning signs in a timely manner. Recognizing the gaps between what is known to improve the outcomes of patients and what is used in daily practice is one of the main objectives of the KT, defined in this case as the change, synthesis and application of knowledge to improve health and provide effective health services [12]. The development of effective KT interventions that maximize the knowledge of clinics and patients about best practices is an important step in closing the gap between what is known and what is practiced.

Considering that the highest proportion of maternal deaths occur during the period of delivery care [5-7], and given the identified need to reinforce and develop strategies to reduce maternal mortality and improve maternal and perinatal health, effective strategies have been implemented focusing on improving the quality of delivery care in health institutions [8], in prenatal care through periodic monitoring by health professionals and in preparation for delivery through the psychophysical course. Strategies of social appropriation of knowledge in health in the latter case have shown that an informed patient, aware of their health care and taking an active role, will have a positive impact, resulting in better health outcomes [9].

Although these strategies have proven to be effective for the fulfillment of these objectives, in Colombia there is a dispersed rural population that has important barriers and limitations for access to health services, especially in attending the psychophysical course that consists of approximately 10 sessions in the case of pregnant women and their caregivers.

It is necessary to generate strategies of social appropriation of knowledge in health focused on the effective communication of basic and relevant information in maternal and neonatal health which reaches this dispersed population, generally located in the quintiles of scarce resources and therefore, as a result of low educational levels, have poor health literacy and who according to the data presented here have the highest MMR.

A program of social appropriation of health knowledge based on evidence, focused on care related to childbirth care and the main warning signs, and addressed to the general population through extracts of key information, disseminated through mass media, would provide the possibility of access to this information for the entire population, particularly impacting the dispersed population with its difficulties in accessing health services regularly, and reinforcing the knowledge transferred in health institutions. The information transmitted in a clear language and in a specific way contributes to the social appropriation of knowledge.

Additionally, the formulation of evidence-based decisions (EBD) depends mainly on clinical expertise and the integration of higher quality evidence, together with information related to patients' preferences, clinical context and resources [2, 10]. The considerations in the search for evidence in the decisions of daily practice are part of the important skills and behaviors for health professionals and are part of the individual accreditation standards [11].

The strategies of knowledge transfer and social appropriation, such as education, reminders and knowledge capsules, have been described as ways to ensure the use of scientific evidence by decision makers or to inform healthcare providers [10].

Among the deficiencies of current health care is the failure to adhere to evidence-based practices for both the prevention and the diagnosis and treatment of diseases. Data from the United States indicate that about 40% of patients do not receive the recommended care and 25% receive unnecessary interventions; this results in the inappropriate use of drugs and devices, increasing morbidity and mortality, as well as the misuse of resources. As such, there are opportunities to improve outcomes and reduce the expenditure of resources by increasing the use of the best available practices as in the case of maternal and perinatal health in our country [1].

Variations in practice are undesirable and lead to unfavorable clinical outcomes, especially in the care of pregnant women and newborns, where in addition the risk associated with errors of omission is high. Maternal and perinatal care faces challenges over time, in terms of adoption and adherence to evidence-based practice. These circumstances lead to underutilization or overuse of therapies. Systemic reviews of KT, which are based on the effective implementation of best practices, have not up to now included maternal care [10, 12].

Translating the best evidence into programmatic information is a complex process [10]. There are multiple barriers to the use of EBD at different levels – for example, the health system (lack of financial incentives), health care organization (limited access to evidence), health care teams (existing standards may be different from the recommended practice), personal preferences of each health professional (lack of knowledge, attitudes and skills, use of evidence, resistance to change), and patient preferences (poor compliance with recommendations) [1,5].

The KT is based on the notion of the quality of care and the improvement of the health of the population when clinical findings and evidence are implemented in daily practice; however, it is not a linear process. Multiple factors, such as the volume of literature, the lack of resources and other individual and institutional issues, make the incorporation of evidence into practice more complex. Research in KT seeks to identify effective interventions that evaluate these barriers and thus improve outcomes in patients. Little is known about the effectiveness of interventions related to KT, especially those related to maternal and perinatal health literacy [9, 10, 12].

Globally, health care systems experience challenges to improve the quality of care and decrease the risk of adverse events [1]; these systems generally fail to use evidence in an optimum manner, resulting in inefficiencies and decreased quality of life [2,3]. For example, McGlynn *et al*. found that less than 55% of adults in the United States received the recommended care [10]. Providing the evidence resulting from health research is necessary, but not sufficient to provide optimal care or make decisions; KT is necessary to respond to these challenges. The growing emphasis in KT has allowed interdisciplinary research fields to be established.

In light of the above, this research intends to answer the questions: (1) What are the characteristics and relevant content in the design of strategies of social appropriation of knowledge in maternal and neonatal security directed to patients and to the community? and (2) What is the effectiveness of 12 knowledge capsules in maternal and neonatal health to achieve social appropriation of this knowledge in the target population?

1. **Justification**

In Colombia, the figures of maternal mortality reported by the Ministry of Health and Social Protection (MSPS), exceed the national targets established in 2003 in the emergency plan against maternal mortality [4]. In 2011, the Health Situation Analysis Report 2013 [13] described an MMR of 68.82 deaths per 100,000 live births; in addition it is reported that maternal mortality in the department of Chocó is 4.2 times higher than the national average, showing the highest number of maternal deaths in the country, with an MMR of 357.97 deaths per 100,000 live births, followed by the Guajira department, with an MMR of 166.85 per 100,000 live births. This statistic, in turn, was shown to be higher in the population located in the poorest quintile, being 1.72 times higher in the departments of the quintile with a greater proportion of Unsatisfied Basic Needs (Vaupés, Sucre, Córdoba, Guainía, La Guajira, Vichada, and Chocó).

Payers, insurers and regulatory bodies increasingly expect the integration of new knowledge with health professionals and scientific evidence in daily practice [1, 2]. This is based on the objectives of the use of evidence-based practice [10], which has shown that it has a direct impact on improving patient outcomes [10].

The KT's interactive models emphasize the nature of the person in the process. That is to say, the transfer is made easier when the producers of the knowledge and the users of it understand each other and are both familiar with the needs, objectives and preferences. Although the importance of familiarity has been documented, it is often weak. Researchers often express frustration with the difficulty of understanding the internal workings in the user groups they evaluate [14].

In order to identify the causes related to the high maternal mortality figures, a systematic analysis developed by the WHO [15] showed that for the period 2003-2009, 73% of all maternal deaths were related to direct obstetric causes, hemorrhage and in particular postpartum hemorrhage being the most frequent direct cause. Hemorrhages, along with hypertensive disorders and sepsis, are responsible for more than half of maternal deaths in the world [15,16]. It is evident then, that the highest proportion of maternal morbidity and mortality occurs during the period of delivery care and the strategies should be directed to intervene in these causes.

In pursuit of this objective, efforts have been made both to improve the quality of care in health institutions and to minimize the patient's risk of undergoing an adverse event. Considering that both surgical units and obstetric units (which include a major surgical component) are the units with the highest number of serious adverse events [17], strategies have been implemented such as the safe surgery checklist which was launched by the WHO in 2008 [18], and having demonstrated its effectiveness, was adapted to the obstetric field as the safe delivery checklist, in order to improve the quality of institutional delivery care, a factor identified as an important contributor in maternal and fetal mortality [19] and whose impact has been evaluated in different countries [20, 21], including ours.

Strategies like these aimed at reducing the number of maternal deaths should be targeted not only at health professionals and institutions. Strategies of social appropriation of knowledge in health that contribute to the empowerment of women and their families to improve and increase the control of maternal and neonatal health, especially in the preparation of childbirth, should be a fundamental pillar in interventions aimed at contributing to the fulfillment of the millennium objectives.

Additionally, based on advances in the knowledge of the relationships between cognitive functions and the nervous system and the possibility of their measurement through neuroscientific methods that allow the establishment of the cerebral and autonomous response to certain visual and auditory stimuli framed within the concept of neuromarketing [22], it provides the possibility of evaluating the retention capacity and the effectiveness of the stimulus issued, which for the present project corresponds to the knowledge capsule in health as a strategy for social appropriation of knowledge.

Therefore, a process of social appropriation of knowledge in health based on evidence is proposed, along with a process of evaluating its effectiveness through neuromarketing strategies, aimed at recognizing warning signs and to know the care and interventions that will be received before childbirth, during the birth and afterwards, for both vaginal births and caesarean, in accordance with the domains included in the safe childbirth checklist. This strategy will contribute to the knowledge and empowerment of the pregnant woman and her family, which will result in better care in the stages of childbirth and postpartum and in better health outcomes for both the mother and the newborn.

The brain of the mother in pregnancy has been studied from multiple perspectives and approaches, both cognitive and emotional as well as neuroendocrine, trying to explain the behavior of the mother in pregnancy, childbirth and postpartum and adding an aspect of neurophysiological complexity to the already complex altered physiological pattern of women in pregnancy [23]. This makes the study, design and management of social transfer and appropriation of knowledge strategies that influence both the pregnant woman and her environment even more important, as an effective strategy in their adherence and transformation capacity for pregnant women, with all the physiological and neurophysiological challenges which motherhood brings. The addition of the neuromarketing strategy in this field is a highly relevant tool in the study of this transfer potential, above all for pregnant women, but also for their environment.

1. **Conceptual framework**

**Knowledge transfer**

KT was defined by a consensus of experts by the World Health Organization (WHO) in 2005, as "the synthesis, exchange and application of knowledge by decision makers to accelerate the benefits of local and global innovation, strengthening health systems and improving the health of people”. The successful transfer of knowledge was conceptualized as a supply or dependent factor (availability of evidence, appropriate packaging, evidence-based messages) and demand or demand factors (local knowledge, political support for the implementation of scientific research, strategic presence of decision makers) [14]. KT barriers are also divided into demand factors such as poor access to relevant tests, production costs, packaging and distribution of technologies necessary for KT [10].

Doctors rarely follow evidence-based guidelines [14, 21]. Likewise, managers and policy makers ignore solid evidence when designing services or allocating resources [10]. Solutions to these problems involve the controlled supply of research evidence or the formation of demand for evidence through education, facilitation, financial incentives or the use of technology adoption channels. KT tries to overcome this challenge through strategies based on three assumptions. The first of these is that "knowledge" is provided by the results of objective and impersonal investigations; that is, it is provided by explicit, consistent and reproducible knowledge. Normally this is shown in the results of randomized clinical trials or meta-analysis and in all cases the knowledge is seen as information that the professionals who generate it can use. The second assumption is that it is useful in KT to conceptualize the "know-how" gap between scientific facts and practice; this implies that knowledge and practice can be separated both empirically and analytically. The third assumption is that the practice consists of a series of rational decisions, in which the results of scientific research can be used as support [10, 12].

These assumptions are widely used in medicine, but as argued below, they are also widely questioned by scholars outside of this field. [14] The "know-how" gap will never be completely closed using "evidence-based concrete action messages" or combinations of incentives in the use of research evidence in clinical meetings. More likely, the construction of a narrower link between knowledge and practice will occur, at least in part, through what Kemmis (a writer in the literature on education) calls "personal praxis," which consists of reflective, individual and collective considerations of how each case and particular situation has been handled (or should be handled). This concept is related to the notion of Lave and Wegner of practice in the community, in which the acquisition of "personal praxis" goes hand in hand with the development of identity and participation of social groups [14].

Gabbay and Le May disputed the assumptions when they carried out ethnographic studies, evaluating the way in which general practitioners use "knowledge in practice within a context" [24]. During their observations they did not find that clinicians consulted any guide to help them make decisions during the approach of a patient in clinical scenarios [10] The doctors used complex and flexible guidelines, which incorporate a mixture of different types of explicit, general and specific knowledge, acquired throughout a lifetime of learning, reading and experience. These mental lines are continually adjusted thanks to written sources but are mainly modified by reflecting on the experience with discussions with colleagues and opinion leaders, sharing stories of real cases, etc. [24].

Eccles *et al*. explain how the theory can be used to help design interventions related to KT and thus understand their impact on individuals and team behaviors. They emphasize two main objectives to apply these theories; firstly, develop factors based on understandable theory, underlying the clinical practice, and identify the theoretical constructs that are important to characterize current models of care. This implies the development of theories that take into account multiple variables that influence clinical behavior, and in this way design appropriate interventions to influence the adoption of desired behaviors by decision makers. The second objective is to develop or test KT interventions whose purposes are specific theoretical constructs and thus design interventions that improve the process of change in these interventions [14]. However, while Eccles *et al*. (2005) recommend the use of systematic theories to increase the probability of successful implementation, these theories are rarely used to inform the design and evaluation of KT interventions [16,17, 5,18].

The review by Davies *et al.* found that only 6% of the included studies used the theory to inform the design and/or implementation of KT interventions. Many of the theories included were related to behavior or behavioral changes, including diffusion and innovation, belief models in health and organizational development. This review identified studies that report KT interventions in two broad categories of theories: cognitive theories and learning theories. None of the studies reviewed theories of social constructivism [14].

Some authors conceptualize KT as a process that occurs in environmental and social interactions, in which the exchange of knowledge between researchers and health care professionals must occur in a mutually created social context [21, 31-33]. In effect, the use of knowledge within the KT can be considered as an active knowledge process, since knowledge is not an inert object that can be sent and received, but a collection of knowledge made up of those that produce it and those who use it. Clinicians act on new knowledge, transforming information based on pre-existing experiences which relates to existing knowledge and monitoring what they understand about the process.

Therefore, the meaning of the research is constructed by users and groups of clinicians as a problem solver and a tool to build their own knowledge, beyond being a passive receptacle of information [22]. This allows us to propose a theory of social constructivism that can be useful to understand why and how individuals internalize and apply their new knowledge in making clinical decisions based on evidence and how practical behaviors can change as a result of targeted KT interventions on the dogmatic core of that theory.

The social context is the center of constructionism and the attention is in the knowledge that is created through a shared production. Constructionism also emphasizes maintaining our culture: this is how we see things and gives us a definitive view of the world.

In contrast, within the paradigm of social constructivism, the individual is the center of the meaning of experience. The center of constructivism is in the individual learning that takes place in the interactions within a particular social context. The fundamental premise of this theory is that the construction of human knowledge and learning is an active teaching process. Constructivism is based on three assumptions about learning. Firstly, learning is the result of individual interactions with the environment: knowledge is constructed, so that the learner gives meaning to his experiences in the world; the content of learning is not independent but is acquired according to the context and therefore the objectives of the receiver of knowledge are involved in the whole process. Secondly, there is a cognitive dissonance that occurs when two opposing thoughts meet at the same time: this is the stimulus to learn, and serves as a force that leads the mind to acquire new thoughts or to modify existing beliefs and thus reduce the amount of conflicts.

These cognitive dissonances determine the organization and nature of what is learned. Thirdly, the social environment plays a critical role in the development of knowledge. Other individuals in the environment may try to test what is understood and give alternative points of view to the learning questions. Constructivism supports the acquisition of cognitive processing strategies, self-regulation and problem solving through constructed learning opportunities. All this is part of critical skills that must be taken into account in evidence-based knowledge and the implementation of clinical practices taking into account actions from the perspective of the KT [25, 26, 28, 30]. Among the structures of this type of action is that adopted by the Canadian Institute for Health Research, which focuses on the creation of exchange and creation of knowledge. This structure contains two main components, a concentration of knowledge creation and a cycle of action. The concentration of knowledge consists of three phases: knowledge research, synthesis of knowledge, and creation of products and knowledge tools.

The action cycle consists of seven stages that involve transferring knowledge into practice: the cycle begins with the identification of a problem in practice or a gap in knowledge; once the problem is identified, a search, identification, review and selection of knowledge is made to implement and act on that gap. This knowledge must be adapted to the local context, evaluating the factors of the use of the knowledge and identifying barriers to its implementation, and selecting and implementing interventions taking into account the previously identified aspects. Subsequently, the use of the knowledge in practice is monitored and the outcomes or impact of the use of new knowledge are evaluated. Finally, strategies are determined to ensure the use of new knowledge [32].

This structure is based on the paradigm of constructivism that favors social interactions and the adaptation of research evidence to take into account local contexts and cultures [34]. Despite the increasing recognition in the framework of the creation of KT of the use of knowledge in practice, these associations with constructivist theories have not yet been explored explicitly.

This social constructivism approaches the science implicit in KT and has the potential to help researchers interested in examining how to learn in a clinical context, and helps understand how new knowledge is created, disseminated, exchanged and used to inform the practical aspect. While theories of social constructivism may be useful in informing the design and evaluation of KT interventions, the degree to which these theories can be applied in the literature used by health care professionals is still not understood.

There are many terms used to describe the process of putting knowledge into action. In the United Kingdom and Europe, the terms of science implementation and use of research are used as common terms in these contexts. In Canada, the terms of exchange and knowledge transfer and KT are commonly used. [5, 6].

Multiple factors determine the use of research in different groups of decision makers [14-18]. A common challenge that all decision makers face is related to the limited skills of the management of knowledge and infrastructure [14, 17]. In a review of the information barriers of medical guides, more than 250 adherence barriers were identified, including lack of awareness, lack of agreement with the guidelines and the presence of external barriers in following the recommendations. Frequently, multiple challenges are presented that operate differently from the health system [14]. It has been suggested that the theory should be used to guide the design of usable intervention strategies, and thus contribute to generating generalized knowledge about the implementation of interventions.

Currently, there are no clear directions to help researchers and practitioners on implementation strategies among the groups of decision makers.

KT has been conceptualized in three frameworks: a focus on linear knowledge transfer; one focused on the KT as a social process; and another that seeks to incorporate contextual issues in the process of understanding the implementation of research.

**Neurocognition and Neuromarketing**

For the brain to assimilate the processed information about its environment, it requires basic cognitive functions. These include *attention*, the main neuronal network for the development of cognitive processes, which regulates the entry of information and directs it to its final cognitive processing; *perception*, which modulates the external information, interprets it and helps its internal representation; *memory*, which contributes to the efficient allocation of information, depending on the attention capacity; *motivation*, related to interest and intentions, and lastly *emotional regulation* [25-27].

The cognitive neuroscience is established as a recent field of science, which unites cognitive psychology, a discipline that studies the higher mental functions, with neuroscience, which has allowed us to understand, through a cellular and molecular approach, that the function of the nervous system is defined by different biological processes [28].

In recent years, the availability of different techniques such as neuroimaging and non-invasive brain stimulation techniques have allowed for better understanding of the relationship between mental functions and the neural systems [29, 30]. Thus the union of these two techniques through cognitive neuroscience is oriented to the understanding of the relationship between the brain and the mind, and tries to answer to how the brain receives, integrates and processes information and how it sends different signals to regulate multiple functions in the body, as well as to solve problems and cognitive processes such as language, attention, or learning and memory mechanisms [31]. Given the relevance of these findings, a particular interest is generated in different scientific and social scenarios and in areas such as marketing and communications.

Neuromarketing is an interdisciplinary field that seeks to evaluate, through neuroscientific methods, the cognitive and emotional responses of consumers to various marketing stimuli, uniting neuroscience, psychology and marketing [32]. Several indicators are measured and evaluated under this strategy, and include emotional commitment, memory and recall, knowledge, attention, and intention to buy. Therefore, the clear formulation and adequate understanding of the message received, as well as the activation of attention and emotion mechanisms, will be able to predict the retention capacity and to a certain extent the effectiveness of the commercial or campaign [22].

Neuromarketing uses different methods that allow the recording of brain electrical activity and metabolic activity. One of the most widely used methods is electroencephalography (EEG), a non-invasive method that detects electrical signals produced by brain activity through sensors. Using the concept that different brain areas are responsible for different functions, depending on the type of stimulus specific cortical areas will be activated [33]. Other techniques that record brain electrical activity, such as functional Magnetic Resonance Imaging (fMRI) and Positron Emission Tomography (PET), are costly methods that limit their application in some way [22].

Another method widely used in neuromarketing makes use of the measurement of ocular dynamics by means of the eye-tracking technique, used to detect and record the activity of eye movements, and establishing during the assignment of a visual task a measurement of the relationship between these movements and cognitive processes such as the processing of language and images, memory processes, social cognition and decision making [34]. The autonomous response of an individual produced by a stimulus is also used in this discipline through the measurement of changes in heart rate and electrodermal reactivity through the galvanic response of the skin, which is related to changes in the sympathetic system associated with emotion, cognition and attention [33, 35].

The knowledge of neuroscience and the study of cognitive functions have also been the basis for the development of cognitive and metacognitive strategies applied to the educational field [36, 37] and which seek to facilitate the different phases of information processing and therefore cognitive performance. These strategies have been designed to strengthen the cognitive processes of attention, understanding and memory. For the attention and comprehension process, the main desire is a narrowing of the attentional focus, which allows us to focus our attention on a single thing, through the focalization of objects, the fragmentation of the visual field in order to better focus and pay attention and ignore distractors. For the memory process, visual techniques have proven to be more effective, considering that images are more easily remembered; verbal techniques are characterized by the use of language as a processing tool, and include mnemonics and keywords [38].

In recent years the knowledge of these cognitive strategies has defined the basis of the design and implementation of some strategies of knowledge transfer towards the patients, in order to generate a suitable use of the new knowledge and a greater adherence to the interventions [12, 39].

1. **Current approaches**

The exchange, synthesis and ethical application of knowledge within a complex system of interactions between researchers and users, aims to accelerate the capture of research benefits through an improvement in health, effective services and products and a stronger health system [1]. As such, it is more concerned with the dissemination of information based on research and moves towards the application of knowledge in practice [2,3]. However, incorporating this type of strategy in a complex and variable system of interactions such as health care requires interventions and strategies of the same level of complexity. Taking this into account, the evaluation efforts focused on added effectiveness would represent a simplification of the interventions and the system of health care interactions itself [10]. Scriven refers to this type of evaluation as a "black box" [10]; the selection and application of the black box or aggregate evaluation may have been influenced by experimental models used to examine the effectiveness of interventions under controlled conditions [10]. In these scenarios, a small number of outcomes are measured based on the anticipated effects of a limited number of variables controlling for confounding effects [10]. However, in terms of effectiveness, in the real world over-simplified measurement models provide little information about the true effect of complex interventions within an uncontrolled scenario, and moreover may not provide information for future study [4, 5, 7, 12].

Approaches based on theory provide an alternative to the "black box" type of evaluation that examines the outcome and takes into account the possible causes and factors associated with the change between interventions [13]. This type of approach can be defined as "any strategy that integrates the use of theory in the conceptualization, design, conduct, interpretation and application of evaluation" [14]. Ideally, it should explain any causal mechanism based on postulated associations between the factors [14]. These types of evaluations may not be associated with any particular ideology or philosophy; that is, it is an evaluation based on explanations that has a generic approach to evaluation, focusing on scientific realism [15], which assumes that reality exists separately from independent perceptions [6, 15, 17, 18] given that its objective is to examine existing models among reality, thus offering a more comprehensive understanding of these models and providing a thorough explanation through the exploration of causal mechanisms, which are sensitive to different types of influences, whether contextual or social [13,16-18]. This type of approach recognizes that it is not possible to reach a complete understanding of reality; however, knowledge is emerging, and contributes in an accumulated way to the understanding of this [17,18].

The intervention mechanisms are not seen as equivalent to the components of the programs; they try to represent the way in which the resources of the programs are received, interpreted and put into action by the participants, which produce one or several outcomes [6, 9, 15].

An evaluation of this type must contain at least a) an explanatory focus, b) a section where grouped configurations of the context, mechanisms and outcomes are investigated, c) the use of multiple methods to collect data.

The evaluation process in reality acts according to a traditional cycle of hypothesis generation, testing and refinement [9,15]; it is suggested that this type of evaluation operates at a mid-range, using concepts and data that range between the description and hypothesis of day-to-day implementation in a universal theory [10]. The conventional measurement of the effectiveness of interventions offers little insight into the mechanisms in a complex learning environment.

The mechanisms of the programs are described as underlying processes, which refers to how interventions modify the outcomes and explain the impact of the program on each individual from the source of the intervention [10]. Regarding the measurement of outcomes, programs or interventions should be tested against a range of indicators for each of the outcomes, both for measurements before and after applying these interventions [9, 19].

Currently there is no measurement representing potential changes in the outcomes of KT in the field of maternal and neonatal health, so it is therefore necessary to look for tools to measure changes at the level of knowledge, understanding or attitudes. Additionally, these should be reasonably associated with potential causal mechanisms within interventions focused on the KT [46].

KT strategies are divided into those that "push" or "pull" knowledge. The strategies that "push" take into account efforts that increase the flow of knowledge between those entities or people that generate knowledge and those who can benefit from it. This involves a process of synthesis of information, as well as efforts for the distribution and promotion of this information. In health care, this type of strategy may involve scientific publications, reports, systematic reviews, clinical practice guidelines, online material, courses, and presentation at conferences, among others.

On the other hand, KT strategies that "pull" knowledge are those in which people seek to obtain new knowledge, in such a way that the entities that provide health care, policy makers or patients actively seek the knowledge they need to make decisions. These efforts can be facilitated with an effective search tool to locate the necessary information, as well as training in the identification and application of research results.

This can be successfully applied with three groups of key decision makers:

1. Allied health care providers and professionals.
2. Patients and the general public.
3. Decision makers.

The structure of the KT developed by Graham *et al*. focuses mainly on the support and promotion of the implementation of scientific evidence, while Kitson *et al*. proposes that there are several types of knowledge that are necessary to develop an effective KT intervention: investigation of evidence, knowledge of the subject and the local context, as well as the experience of the participants [40].

The objective of research in KT is to develop theoretical bases to optimize knowledge. The field of research in KT is growing, but still presents methodological and conceptual challenges. The interventions in KT generally have a complex design, with equally complex evaluations [11].

International organizations have implemented strategies aimed at health institutions and professionals, with tools such as evidence-based clinical practice guidelines, institutional management protocols and the safe delivery checklist, with the purpose of reducing variability in clinical practice and improving the quality of delivery care. Likewise, strategies for the standardization of clinical practices in health have been developed, such as clinical practice guidelines in versions for patients and programs that highlight the importance of early prenatal control and preparation for childbirth. These guides seek to emphasize the use of existing evidence and theory throughout the process of childbirth. In addition, research based on KT theory provides benefits such as the development of a generalized framework to measure barriers, facilitators and optimization of the selection of the components of the interventions, as well as the design of a strategy for evaluating them [11].

An informed and empowered patient has even been shown to improve patient safety and constitutes a safety barrier in the presence of adverse events [41].

Considering the findings described above, and in view of the high figures of maternal deaths that still occur in our environment, in 2010 the WHO created the “Making Pregnancy Safer” (MPS) department. This department states that in order to reduce maternal mortality, not only the improvement of health services is required, but also collective participation, which contributes to the empowerment of women, families and communities to improve and increase the control of maternal and neonatal health, as well as to increase access to quality health services [42].

This project therefore intends to develop knowledge capsules in maternal and neonatal health based on the theory of KT and to evaluate its potential effectiveness in order to achieve social appropriation of knowledge, based on neuromarketing tools.

1. **Objectives**
   1. **Overall objective**

Design and evaluate the effectiveness of 12 knowledge capsules in maternal and neonatal health through a pilot study to generate knowledge appropriation in the target population.

1. **Specific objectives**

- Determine the effectiveness of different strategies of social appropriation of knowledge through a systematic review of the literature.
- Based on scientific evidence and expert opinion, design 12 capsules of knowledge in maternal and neonatal health.
- Evaluate the effectiveness of the capsules in maternal and neonatal health in terms of social appropriation of knowledge, measured from attention, emotional response and recall through a pilot study, applying neuromarketing strategies.
- Produce a final version of the knowledge capsules in maternal and neonatal health with the design and content defined in the previous phases.

1. **Methodology**

The development of the project will be developed in 6 phases:

1. Systematic review of the literature.
2. Preliminary definition of the contents of the knowledge capsules in maternal and neonatal health.
3. Formal consensus of experts under the modified Delphi method where the content and strategies to be used in them will be defined.
4. Design and development of health capsules in maternal and neonatal safety.
5. Evaluation of the effectiveness of the knowledge capsules in maternal and neonatal health through a neuromarketing strategy, with a crossover experimental study.
6. Final production of health knowledge capsules in maternal and neonatal safety
7. **Phase 1. Systematic review of the literature and meta-analysis**

***- Design of the study:*** A Systematic Review of Literature (RSL) will be carried out, with the objective of reviewing and critically evaluating the literature on the effectiveness of different strategies in the transfer and social appropriation of knowledge in health. This RSL will include randomized clinical trials. In the event that this level of evidence is not found, we will proceed to look for cohort studies that evaluate this type of interventions in the general population.

***- Research question:*** the orientation of all steps in the development of the RSL, are within the framework of the following question:

In pregnant women, what is the effectiveness of KT strategies in health that include audiovisual methods, compared with other similar strategies (written material, talks with health personnel, etc.)?

***- Methodology***

**- Eligibility criteria**

*Inclusion criteria*

Population: studies in which the general population participates.

Intervention: Studies whose objective has been to implement KT strategies in health that include audiovisual methods.

Comparator: studies whose objective has been to implement other KT strategies in health (written material, talks with health personnel, etc.).

Primary Outcomes:

− Health outcomes

− Quality of life

Secondary outcomes:

− Use of health services

− Costs, cost effectiveness

− Level of knowledge on the subject after the intervention

Studies

− Publication format: studies available as a complete publication.

− Language of publication: English or Spanish.

− Publication status: published studies, in press or gray literature.

− Publication date: without restriction

−Design: randomized clinical trials and observational studies that evaluate the effectiveness of different KT strategies in health.

*Exclusion criteria:* None.

**− Evidence search**

A systematic and exhaustive search of literature will be carried out.

*− Search in electronic databases*

To identify indexed publications, the following sources will be consulted:

− MEDLINE (Ovid platform)

− EMBASE (Ovid platform)

− LILACS (Virtual Health Library - VHL, iAHx interface)

− Cochrane Central Register of Controlled Trials – CENTRAL

− WHO International Clinical Trials Registry Platform ICTRP portal

− PsycINFO

− CINAHL

The search strategy will be composed of controlled vocabulary (MeSH, Emtree and DeCS) and free language. It will be complemented with expansion of controlled terms, truncators, proximity operators and Boolean operators to look for variations between the terms.

Searches will be conducted without language restriction.

**− Complementary search methods**

A "snowball" search will be conducted by reviewing the list of bibliographic references of the selected studies.

For each search a report will be generated, which will include the specific search strategy for each database, along with its results. Subsequently, duplicate references will be eliminated through the EndNote X6® program.

**− Screening of references and selection of studies**

Two reviewers will independently filter the references by their title and summary format and subsequently those that meet the defined inclusion criteria will be selected, after reviewing the full text of the publication. Any disagreement between the reviewers will be discussed as a group and a consensus will be reached regarding the inclusion or not of the studies.

**− Evaluation of the risk of bias of the selected studies**

The risk assessment of bias in the selected studies will be carried out using the bias risk tool of the Cochrane Collaboration for randomized clinical trials and using the SIGN tool for the identified cohort studies.

**− Assessment of the quality of the evidence set**

The quality of the whole of the evidence will be evaluated following the GRADE methodology and tables of evidence will be generated with the results obtained for each outcome found.

**− Data extraction and synthesis of the evidence**

The characteristics of the selected studies will be summarized using standardized formats. The data to be extracted in each study are:

- Study ID
- Year of publication
- Type of educational tool used
- Comparator
- Evaluated outcomes
- Results

**− Statistical analysis**

This will be done independently, both for randomized clinical trials and for cohort studies. In the case of clinical trials, the plan is to perform a statistical combination of the results of two or more individual studies identified through a meta-analysis, after screening for the presence of heterogeneity through the inconsistency test (I2), and the p value of the chi-square test, and if there is variation in the definition of the evaluated outcomes, statistical heterogeneity will be considered if an I2 greater than 40% or a p less than 0.10 is found in the chi-square test. The statistical combination of the results will be done through a meta-analysis of fixed effects or random effects if the data are heterogeneous. A sensitivity analysis will be carried out to study the influence of the risk of bias in the results of the meta-analysis. The presence of publication bias will be evaluated by means of a funnel plot and Egger's statistical test.

In the case of the cohort studies, the results of each outcome will be presented in the units of measurements reported in each study.

1. **Phase 2. Preliminary design of the contents of knowledge capsules in maternal and neonatal health**

Based on the results of the RSL, and following the methodologies described in the evidence for the development of the most effective KT strategies in health, the preliminary contents of the capsules will be designed, principally considering the key information for the preparation of the delivery and post-partum care which must be transmitted to pregnant women, which allows the generation of an adequate social transfer of knowledge. These contents will be defined in accordance with the domains described in the safe delivery checklist and will be designed for pregnant women who have a vaginal delivery as well as those who have a cesarean delivery.

These contents will be described independently, for the pre-partum, intra-partum and post-partum phase, and will include aspects related to patient safety, warning signs, interventions that will be received during delivery care and aftercare, both for normal delivery and for after surgery. It is proposed to make 2 capsules for each phase, one for each of the two possible means of delivery, for a total of 12 health capsules. The preliminary design of the 12 capsules will be carried out by the researchers of the group, and the proposal will be presented to the external experts in Phase 3.

1. **Phase 3. Formal expert consensus under the modified Delphi method**

The Delphi method is a structured consensus methodology that gathers and processes the opinions of experts on a specific problem, and by means of statistical resources builds a general group agreement [43]. This phase of formal consensus aims to validate the relevant contents of the 12 capsules with the experts.

*Sample size:* In this case, the sample size does not depend on the statistical power, but on the dynamics to reach a consensus within the panel. A minimum of 10 experts is recommended [44]. Ten experts will be convened in gynecology and obstetrics, psychology and health education, promotion and prevention programs, with at least 5 years of experience in maternal and perinatal health.

*Preparatory phase*

The members of the panel will be selected according to the previously defined profiles. A document synthesizing the identified evidence and the results of the RSL and meta-analysis will be prepared and the corresponding questionnaire will be designed for the consultation phase.

*Consultation phase*

In a first face-to-face meeting, the summary of the identified evidence will be presented to the panel of experts, as well as the preliminary content of the health capsules developed in the previous stage of the project.

Two additional consultation rounds will be held; for each of them, the statistical analysis will be performed, which will correspond to the description of measures of central tendency and dispersion and measures of position such as the median, based on a Likert scale that will be designed for that purpose [45].

After the rounds of consultation and statistical analysis, feedback on the processing of the results will be carried out.

*Consensus phase*

− Consensus building: a general group agreement or consensus will be defined, when there is a minimum agreement of 80% between the members of the panel.

− Report of results: A final report will be made that includes a detailed description of the process for each of its phases, the results of the statistical analysis and its interpretation, the difficulties presented during the process and the final result of the panel of experts.

1. **Phase 4. Design and development of health capsules in maternal and neonatal safety**

Based on the results of the systematic review of strategies for the transfer and social appropriation of knowledge, on the results of formal consensus of experts, and on the theoretical foundations of neurocognition and neuromarketing, the design of health knowledge capsules will be developed together with experts in health psychology, promotion and prevention in health, as well as experts in diagramming and development of audiovisual products. During this phase the final scripts, the graphic outline of the clips, their duration, the soundtrack and the production will be created, based on the results of the previous phases.

1. **Phase 5. Evaluation of effectiveness of knowledge capsules in terms of social appropriation of knowledge.**

A neuromarketing strategy will be applied in the Cognitive Neuroscience and Communication Laboratory of the National University of Colombia, with the objective of evaluating the effectiveness of the knowledge capsules in maternal and neonatal health in terms of social appropriation of knowledge, using measurements of attention, emotional response and remembrance.

The analysis will be conducted to find differences between subgroups or categories of educational level, defined as follows:

0 = No study

1 = Primary or Baccalaureate (basic education or secondary education)

2 = Higher Education (undergraduate and postgraduate)

*- Design:* Cross-over experimental study in which a group of pregnant women, non-pregnant women and adult men will be exposed to 13 clips, 12 of which will be produced through this knowledge transfer strategy and one will be an unrelated standard video that will be used as a control. The order of viewing the videos will be assigned randomly for each subject, so that each subject will have 13 evaluations. The random allocation tables will be generated by computer, and one will be made for each educational stratum (without education, with primary or secondary education, and with post-secondary education) and group (pregnant woman, non-pregnant woman and man). It is expected to have a minimum sample of 150 subjects.

*- Population and sample:*

- Inclusion criteria:

Pregnant women, non-pregnant women and men over 18 years of age, who agree to participate in the study through informed consent.

- Exclusion criteria:

participants who presented serious health problems linked to pregnancy or who presented any clinically significant visual and auditory problems, which affected the viewing of the videos.

*- Type of sampling:* not probabilistic, for convenience.

*- Sample size:*

Using the statistical analysis program for biomedical, social and behavioral sciences G * Power 3, the sample size was calculated for a one-way analysis of variance (ANOVA) design and fixed effects, using the F statistic. The selection of the parameters was made for the recall outcome, expecting that the differences of the capsules compared with the video control would be moderate. The parameters were therefore defined as follows: moderate effect size f = 0.3; power (1-β) = 0.90 and alpha (α) = 0.05 for three groups. The calculation yielded a result of 144 for the total sample. Considering that there would be no follow-up of the participants and the measurements would be taken in a single session, a low number of losses was estimated (mainly due to failures in the registry), so a minimum of 150 subjects was defined as a total sample, 50 for each group.

*Process:*

All participants will sign an informed consent (Annex 1) after explaining the objectives of the study and procedures that will be carried out. Participation will be completely voluntary.

The procedure consists of the projection of 12 health knowledge capsules of approximately 35 seconds each, and a control clip, not related to the subject. The control clip will be a standard video, which provides information on reproductive health and maternal safety, but which has not been designed based on evidence or following strategies based on neuromarketing.

The observation of the videos will be free; that is, they will not be explained to the participants during the screening, or the content of them. All the videos will be seen by the participants, but the observation of each one will be assigned in a random way.

The successive order of visualization of the videos is presented in Figure 1. It shows the successive ordering of the clips. However, the actual order of visualization of the clips will be done randomly for each of the subjects.


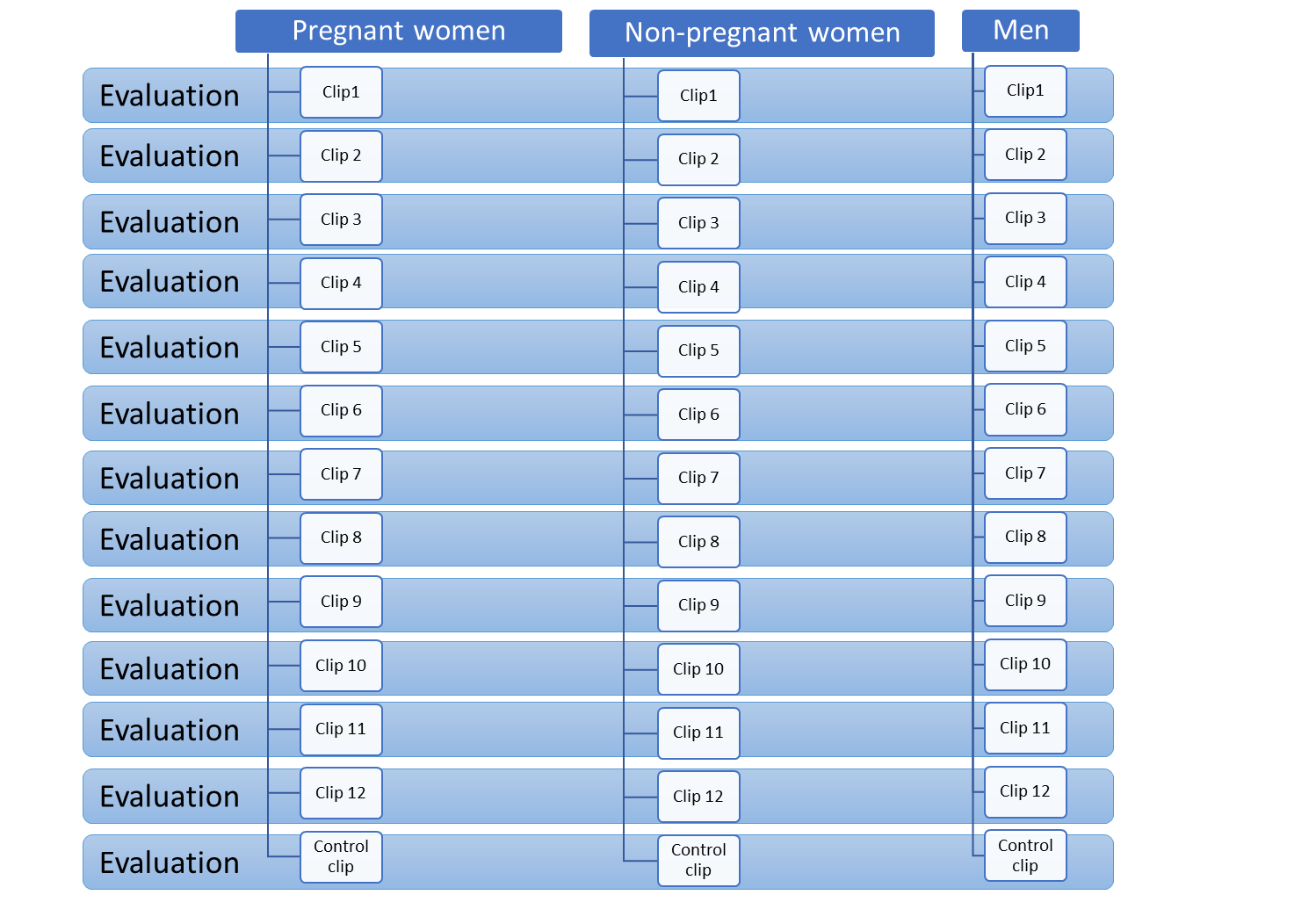


***Figure 1.*** *Form in which the assignment and evaluation of the effect of each intervention is planned.*

The process of consolidating results will be done for each clip as shown in Figure 2.

***Figure 2.*** *Selected sample, and methodological format of analysis of the clips*

During the screening of the health knowledge capsules, the eye tracking method will be applied to evaluate the attention of the subjects before the visual stimulus of the health capsules. By means of a Tobii TX 300 eye tracker and the Tobii Studio Software, the pupillary size in arbitrary units (AU), and the duration and direction of the fixation in seconds will be measured. Areas of interest will also be recorded, according to the stimuli in which the attention is fixed, the duration, and the order in which the visual exploration is performed.

The monitoring of the autonomous activity will be carried out through the registration of the electrodermal activity (Galvical Skin Reaction), which allows the evaluation the state or emotional reaction of the subjects. The record of electrodermal activity will be measured by the computerized psychophysiological recording equipment I-330-C2 + 12. (J & J Enterprises Seattle, WA). The measuring electrodes will be placed on the distal phalanx of the third and fourth finger of the non-dominant hand with SE-35 sensors connected to a T-601 preamplifier (J & J Enterprises Seattle, WA). For the analysis of results, the USE3 Software from Physiolab will be used (J & J Engineering, 2004).

The analysis of skin conductance data will be carried out with a procedure performed by Benedek and Kaernbach, through which the amplitude of the electrodermal response to a stimulus is obtained. Thus, two register values ​​will be recorded to calculate the amplitude: the conductance recorded in the lowest time of the latency period and the conductance peak. Subsequently, these values ​​and their registration will be subtracted. This analysis will be performed for each event identified in each capsule by each subject, to obtain an average of the conductance amplitudes for each grouping of images-events (positive, negative and neutral).

Between each video there will be a break of 5 to 10 seconds, and before starting the next video for evaluation there will be a neutral sequence in order to calibrate the response of the individual to a neutral state: this is considered to be the washout period between each video.

A parametrization of the measurements will be carried out in order to be able to relate both variables. Subsequently, a survey will be applied to evaluate the recall component.

***Statistic analysis:***

Hypotheses raised:

- **Study hypothesis 1**

Comparison between capsules and video control

H0: μ capsule X = μ video control

Ha = μ capsule X ≠ μ video control

*μ = average attention, emotional reaction and recall*

- **Study hypothesis 2**

Comparison between groups: men, non-pregnant women, pregnant women

H0: μ1 = μ2 = μ3

Ha: ∃μj ≠ μ j = 1, 2, 3

*μ = average attention, emotional reaction and recall*

- **Study hypothesis 3**

Comparison between levels of schooling: none, primary, baccalaureate, undergraduate, postgraduate

H0: μ1 = μ2 = μ3

Ha: ∃μj ≠ μ j = 1, 2, 3, 4, 5

*μ = average attention, emotional reaction and recall*

- **Study hypothesis 4**

Comparison between family history: children, no children

H0: μ1 = μ2

Ha: μ1 ≠ μ2

*μ = average attention, emotional reaction and recall*

A descriptive analysis of the measured variables will be carried out. The qualitative variables will be described in proportions, and calculations for averages and standard deviation that follow a normal distribution will be performed for quantitative variables. For variables that do not follow this distribution, medians and interquartile ranges will be calculated. The normality of the variables will be evaluated by the Shapiro-Wilk test.

The difference between the averages of the variables of emotional reaction and attention will be carried out by means of an analysis of variance (ANOVA) for dependent samples, taking into account the effect of drag. For these comparisons, the non-parametric tests of Kruskal Wallis and the Wilcoxon rank sum will be used, according to the number of levels of each factor.

For the results of the survey, in which the recall of the contents of the capsules will be evaluated after their screening, an analysis will be made to compare the difference in proportions between the groups with a good level of recall and those with a low level of recall for each category of educational level.

Statistical software Stata 13.1 will be used to analyze the data.

1. **Phase 6. Final production of the health capsules in maternal and neonatal safety**

Professional experts in the layout and development of audiovisual animation products will carry out the final production process, taking into account the changes suggested by the analysis of results of the neuromarketing strategy.

- - - 1. **Ethical considerations**

The current project adheres to the ethical principles for medical research in human beings, promulgated by the Declaration of Helsinki of the World Medical Association [46] and in the resolution 008430 of 1993 of the Ministry of Health [47], which establishes the scientific, technical and administrative standards for health research. According to this resolution, this investigation is classified as having minimal risk, considering that it uses the data registry through standard procedures and the behavior of the subjects will not be manipulated. For this investigation, a signature of informed consent will be requested for the phases that require it. The entire project will be submitted for approval by an Ethics Committee.

1. **Confidentiality**

The information collected during the development of the project will be restricted to the research team; the information in digital media and databases will have access codes and the information in physical form will be stored on shelves under lock and key for 10 years.

1. **Expected results of the investigation**

After the research is completed, there will be 12 health capsules, designed using neuromarketing strategies, for the preparation of childbirth, both for vaginal delivery and cesarean delivery and the evaluation of their effectiveness in social appropriation of knowledge.

It is hoped that the research, through this strategy of KT, contributes to the knowledge of relevant and clear information for pregnant women and their families and to the empowerment of patients in maternal and neonatal health care. In the medium and long term it is expected that these capsules will be used to be disseminated in a variety of mass media and thus contribute to improve the knowledge and empowerment of the pregnant woman and her family, and in this way the intervention contributes to the improvement of the health and safety of these patients, with a lower proportion of obstetric complications related to pregnancy, childbirth and postpartum.

The project will contribute to the development of the lines of research in maternal and neonatal health and patient safety at the National University of Colombia and the Colombian Society of Anesthesiology and Resuscitation (S.C.A.R.E.).

Once the present work is completed, a second stage will be proposed in which the implementation of knowledge capsules in maternal and neonatal health will be carried out through mass media and the evaluation of their impact on maternal morbidity and mortality.

1. **Expected products**

Results of activities of Generation of New Knowledge

| **Product** | **Description** | **Number** | **Target result/product** |
| --- | --- | --- | --- |
| Knowledge transfer tool | Health capsules for preparing for childbirth, either natural or Cesarean. | 12 | General population, principally pregnant women  Health institutions, national scientific community, Equality in Health Group, Quality, Safety and Health Education Group, Neurosciences Group |
| Original article in national journal | Original article 1: Systemic literature review to evaluate the effectiveness of different KT health strategies. | 1 | Health institutions, national scientific community, Equality in Health Group, Quality, Safety and Health Education Group, Neurosciences Group |
| Original article in international journal | Original article 2: Evaluation of the effectiveness of social acceptance of maternal and neonatal through neuromarketing strategies. | 1 | Equality in Health Group, Quality, Safety and Health Education Group, Neurosciences Group |
| Master’s degree thesis | Evaluation of the effectiveness of social acceptance of maternal and neonatal through neuromarketing strategies. | 1 | Equality in Health Group, Quality, Safety and Health Education Group, Neurosciences Group |

Products results of activities of Strengthening the scientific community

| **Type of result** | **Expected result** | **Number** | **Beneficiaries** |
| --- | --- | --- | --- |
| Links with master’s degree student | Start training of a master’s degree student within the project framework | 1 | Laura Catalina Prieto Pinto, with  CvLAC connection |
| Others | Educational tools for the patient to be applied by health personnel. | 12 | Health institutions, gyno-obstetric professionals |
| Others | Strengthening of the scientific capacities in the development of research projects. | 3 | Equality in Health Group, Quality, Safety and Health Education Group, Neurosciences Group |

Products results of Social Transfer of Knowledge

| **Product** | **Description** | **Number** | **Beneficiaries** |
| --- | --- | --- | --- |
| Scientific event | International congress | 1 | National scientific community, Equality in Health Group, Quality, Safety and Health Education Group, Neurosciences Group, National University |
| Scientific event | National congress | 1 | National scientific community, Equality in Health Group, Quality, Safety and Health Education Group, Neurosciences Group, National University |

1. **Studies of the research team**

S.C.A.R.E.'s studies in patient safety and its efforts in obstetrics has been directed through the research group "Quality, Safety and Health in Education" which, supported by the society's research area, has carried out multiple projects of research on safety in the area of ​​obstetrics, including "gynecology and occupational responsibility claims" carried out between 2004 and 2005, and "adverse events in gynecology" that in 2009 studied the patterns of conduct in medical practice in obstetrics to find out about these activities and generate measures of patient safety, and "Processes of care during pregnancy until the first week after delivery of women with identified risk factors attending the Rafael Calvo Maternity Clinic in the city of Cartagena" (2013). These investigations, as well as the rigorous revision of literature during recent years, have led to products such as the article "Obstetrics-Gynecology and the demands of professional responsibility in Colombia" published in the Medical-Legal Journal in 2005, the book chapters "New guidelines for Cardiopulmonary Resuscitation in pregnant women" published in the XIV Update Course in Gynecology and Obstetrics in 2006, and "Analgesia and anesthesia for the obstetric patient" published in 2004. Additionally, sustained work has been carried out on the checklists and their effectiveness which has resulted in the academic articles "The checklist: a standard of care" and "Verification of the checklist for safety in surgery from the patient's perspective" published in the Colombian Journal of Anesthesiology in 2013, the presentation "Safety in ambulatory surgical patients and experience of the checklist" presented at the National Congress of Anesthesiology in 2011, and the poster “Is the checklist justified as a strategy of the safety policy in the ambulatory surgical unit?”

In addition to investigative interests, the S.C.A.R.E. and the research group have endeavored to implement intervention actions. Among the most notable during the last three years, specifically on safety in obstetrics, are the workshops of Colapso Materno and ALSO (Advanced Life Support in Obstetrics) for professionals and BLSO (Basic Life Support in Obstetrics) for midwives and nursing assistants, all three aimed at the management of obstetric emergencies. These workshops have been offered free of charge in some of the regions of the country with the greatest needs in maternal care, including Guajira, Córdoba, Magdalena and Chocó. This same year in March a national training session was also held in Medellín for trainers in safe anesthesia for obstetrics and management of the main causes of maternal death, in partnership with the World Federation of Anesthesia Societies (WFSA) and the British Association of Anaesthetists (AAGBI).

Within the dissemination strategies of knowledge beyond the academic areas are included the distribution of booklets for the implementation of the checklist and for the management of obstetric emergencies and the disclosure of topics in patient safety in social networks, blogs, forums and news.

For their part, the Equity in Health groups and the Health Policy and Technology Evaluation Group have developed the following studies related to patient safety and maternal morbidity and mortality:

1. Gaitán-Duarte, HG; Gómez Sánchez, PI; Eslava-Schmalbach, JH. Actitudes del personal a la Vigilancia de Eventos Adversos intrahospitalarios en Colombia. Revista De Salud Pública. 2009; 11(5). 745 – 753.
2. Gaitán-Duarte, HG; Gómez Sánchez, PI. Association between pregnancy-induced hypertension and post-partum infection in the Instituto Materno Infantil, Bogotá. Case control Study. Revista Colombiana De Obstetricia Y Ginecologia. 2004; 55(3): 193 – 200.
3. Gaitán Duarte. HG; Magpie trial collaborative group. Do women with pre-eclampsia, and their babies, benefit from magnesium sulphate? the magpie trial: a randomised placebo-controlled trial. Lancet. 2002. 359: 1877 – 1890.
4. Gaitán, HG. Gómez, PI. Escandón, I. Evaluación de la atención integral posaborto en 13 hospitales en Colombia. Revista de salud pública. 2007. 9: 241 – 252.
5. Gaitán Duarte, HG, Daly, S. Chipato, T, Tolosa, J, Festin, M. Lumbiganon, P. Limpongsarunak, S. International survey on variations in practice of the management of the third stage of labour. Bulletin of the world health organization. 2003. 81(4): 286 - 291
6. Gaitán Duarte, HG, Rubio Romero, JA, Gómez, M. Interpretación del desempeño operativo de las pruebas de tamizaje y de diagnóstico en enfermedades en obstetricia y ginecología. Revista colombiana de obstetricia y ginecología. 2009. 60(4): 365 – 376.
7. Gómez, PI; Gaitán, HG. Is there association between preeclampsia and puerperal infection? Case control study international journal of gynecology &amp; obstetrics. 2003.83 (3): 50 – 51.
8. Gaitán Duarte, HG, Ángel Müller, E. Díaz, AL. Revisión uterina como factor de riesgo para endometritis postparto. Revista colombiana de obstetricia y ginecología. 1998. 49: 153 – 156.
9. Gaitán Duarte, HG. Garzón, g. Eslava Schmalbach, JH. Rubio Romero, JA. Forero, J. Screening of adverse events (ae) in obstetric attention and puerperium at the Instituto Materno Infantil, Bogotá, Colombia, 2002-2003. Revista colombiana de obstetricia y ginecología. 2005. 56(1): 18 – 27.
10. Gaitán Duarte, HG. Tamizaje de eventos adversos en atención obstétrica y del puerperio en el instituto materno infantil de Bogotá, Colombia. 2002 -2003. Revista colombiana de obstetricia y ginecología. 2005. 56(1): 18 – 27.
11. García Ulloa, A. Navarro Vargas, JR. Eslava-Schmalbach, JH. Encuesta sobre código rojo en cinco instituciones de salud de Bogotá. Revista colombiana de anestesiología. 2010. 38(1): 51-65.
12. Eslava-Schmalbach, JH. Gaitan HG. Restrepo, CG. Escala para medir la calidad de la recuperación postanestésica desde la perspectiva del usuario. Revista de salud pública. 2006. 8(1): 1 págs: 52 – 62.
13. Fajardo Rodríguez, H. Quemba, J. Eslava-Schmalbach, JH. Escalas de predicción e infección de sitio quirúrgico en 15625 cirugías, 2001-2003. Revista de salud pública. 2005 7(1): 89 – 98.
14. Buitrago Gutiérrez, G. Fitzgerald Arias, G. Amaya Santiago, H. Avila, C. Saavedra Trujillo, CH. Estudio observacional de infecciones nosocomiales diagnosticadas en un hospital universitario de tercer nivel entre los años 2002 y 2003. Infectio. 2004. 8(2): 132 – 132.
15. Eslava-Schmalbach, JH. Gaitán, HG, Prada, L. Alfonso, C. Otálora, W. Factores asociados a la estancia hospitalaria en instituciones de segundo y tercer nivel en Santafé de Bogotá. Investigaciones en seguridad social y salud. 2001. 3: 8 – 44.
16. Gaitán, HG. Eslava-Schmalbach, JH. Rodríguez Malagón, MN. Forero Superlano, VH. Santofimio Sierra, D. Altahona, H. Incidencia y evitabilidad de eventos adversos en pacientes hospitalizados en tres instituciones hospitalarias en Colombia, 2006. Revista de salud pública. 2008. 10(2): 215 – 226.
17. Yomayusa, N. Gaitán Duarte, HG. Suarez, IC. Ibáñez Pinilla, M. Hernández, P. Álvarez, C. Sosa, M. Altahona, H. Arango, A. Zusman, O. Validación de índices pronósticos de infección de sitio quirúrgico en hospitales de Colombia. Revista de salud pública. 2008. 10(5): 744 – 755.
18. Rubio Romero, JA. Guevara Cruz, OA. Gaitán Duarte, HG. Validez de la estimación visual como método diagnóstico de la hemorragia postparto severa en un hospital universitario. Revista de la facultad de medicina. 2010. 58(3): 173 – 184.
19. Eslava Schmalbach, JH. Saavedra Trujillo, C. Quemba, J. Porras, N. Velasquez, O. Zabala, R. Wilches J. Vigilancia epidemiológica de infecciones inthahospitalaria en la clínica Carlos LLeras Restrepo del seguro social. Boletín epidemiológico distrital. 2001. 6 (7-8): 1 – 24.
20. Hull, L.; Arora, S.; Amaya Arias, A.C.; Wheelock, A.; Gaitán- Duarte, H.; Vincent, C., et al. Building and Strengthening Global Capacity for Patient Safety: A Training Programme Incorporating the WHO Core Competencies for Patient Safety Research. Int J Surg. 2012; 10(9):493-499.
21. Otálora, W., Eslava, J. Revisión de Literatura para la Administración de Hospitales Públicos. Investigaciones en Seguridad Social. Secretaria Distrital de Salud, 2001; 3: 139-173
22. Díaz., J. Eslava, J. Estudios de Evaluación Económica de la Tecnología en Salud. Revista Facultad de Medicina, Universidad Nacional de Colombia 2001, Vol 49 (2); 115-118.
23. Eslava, J., Saavedra C., Quemba, J., Porras, N., Velásquez, O., Zabala, R., Wilches, J., Yepes, J. Vigilancia Epidemiológica de Infecciones Intrahospitalarias en la Clínica Carlos Lleras Restrepo del Seguro Social (CCLLR) en Bogotá. Boletín Epidemiológico Distrital. 2001, Vol. 6 (7-8):1-24.
24. EngenderHealth. *Minilaparotomy for female sterilization. An illustrated guide for service providers.* Contribution to the guide with the chapter of Anesthesia (chapter 5): EngenderHealth, New York, 2003, pp.25-38. Tomado de [www.engenderhealth.org](http://www.engenderhealth.org)
25. Buitrago G, Eslava JH, Amaya H, Avila C, Saavedra CH. Estudio de cohorte para determinar la incidencia de los desenlaces clínicos de la infección nosocomial y los factores relacionados a la resistencia bacteriana, en un Hospital Universitario de tercer nivel durante los años 2002 y 2003. Infectio. 2004;8(2):73. ISSN: 0123-9392
26. Eslava J, Gaitán H, Pedraza N. Edema pulmonar en cirugía laparoscópica. Presentación de un caso y revisión sistemática de la literatura. Revista Colombiana de Obstetricia y Ginecología 2005, 56(4): 294-302
27. Garcia I, Valenzuela E, Saavedra C, Leal A, Eslava J, Mantilla J. Caracterización molecular de aislamientos de *Enterobacter cloacae* multiresistentes productores de beta-lactamasas provenientes de pacientes de un hospital de tercer nivel de Bogotá. Rev. Fac. Medicina, 2005, Vol 53 (3): 148-159
28. Escobar F, Folino J, Eslava J. Calidad del sueño en mujeres víctimas de asalto sexual en Bogotá. Rev. Fac. Medicina, 2006, 54(1): 12-23
29. Escobar F, Folino J, Eslava J. Alteraciones del sueño y estrés postraumático en mujeres víctimas de asalto sexual. MedUNAB 2006, 9:28-33
30. Leal AL, Eslava-Schmalbach J, Álvarez C, Buitrago G, Méndez M y GREBO. Canales Endémicos y Marcadores de Resistencia Bacteriana, en Instituciones de Tercer Nivel de Bogota, Colombia. Rev. Salud Pública. 2006, 8(Suppl.1): 59-70
31. González-Mejía E, Valenzuela E, Mantilla-Anaya J, Leal-Castro A, Saavedra C, Eslava-Schmalbach J, Sierra-Rodríguez S. Resistencia a Cefepime en Aislamientos de *Enterobacter cloacae* provenientes de hospitales de Bogotá, Colombia. Rev. Salud Pública. 2006, 8(2): 191-199.
32. Murillo-Rojas O, Leal-Castro A, Eslava-Schmalbach J. Uso de antibióticos en infección de vías urinarias en una Unidad de Primer Nivel de Atención en Salud, Bogotá, Colombia. Rev. Salud Pública. 2006, 8(2): 170-181.
33. Galván-Villamarín JF, Bernal Torres Fabio A, Páez JM, Acero Moreno W, Eslava-Schmalbach J, Calixto LF. Evolución clínica y radiológica de pacientes intervenidos de artroplastia total de cadera con prótesis de resuperficialización metal-metal. Reporte preliminar. Ortopedia y Traumatología. 2006. 20(4) : 96-103
34. Buitrago G, Alvarez CA, Eslava JH, Leal AL, Cortes JA, GREBO. Tendencias de marcadores de resistencia bacteriana En 21 Instituciones de tercer nivel de Bogotá, Colombia, 2001 - 2005. Infectio. 2006;10(2):119-20. ISSN: 0123-9392
35. Eslava-Schmalbach J, Alfonso H, Oliveros H, Gaitan H, Agudelo C. A new Inequity-in-Health Index based on Millenium Development Goals: methodology and validation. J Clin Epidemiol. 2008 Feb;61(2):142-50.
36. Rincon D, Valero JF, Eslava-Schmalbach J. Construcción y validación de un modelo predictivo de hipotermia intraoperatoria. Rev. Esp. Anestesiol. Reanim. 2008; 55: 355-359
37. Navarro Vargas R, Eslava Schmalbach J, Tejada E. Dolor Agudo Postoperatorio Obstétrico y Ginecológico. En Gómez Sánchez PI, Hernández Castro JJ, Dolor en la Mujer. Asociación Colombiana para el Estudio del Dolor. Editora Guadalupe Ltda. Bogotá, 2008. pp. 55-72, ISBN: 978-958-44-4026-6
38. Navarro Vargas R, Eslava Schmalbach J, Gómez Sánchez PI. Dismenorrea. En Gómez Sánchez PI, Hernández Castro JJ, Dolor en la Mujer. Asociación Colombiana para el Estudio del Dolor. Editora Guadalupe Ltda. Bogotá, 2008. pp. 153-161, ISBN: 978-958-44-4026-6
39. Eslava-Schmalbach, J. ¿Anestesia basada en el cirujano o en el paciente?. Editorial. Revista Colombiana Anestesiología. 2008; 36(3).p.p.157-159
40. Henríquez DH, Alvarez CA, Leal AL, Eslava JH, Buitrago G, Cortes JA, Castillo JS. Efectividad de una intervención activa para mejorar la notificación de un sistema de vigilancia en resistencia bacteriana, Bogotá (Colombia). Infectio. 2008;12(S1):111. ISSN: 0123-9392
41. Eslava-Schmalbach J, Buitrago G. La medición de desigualdades e inequidades en salud. Revista Colombiana de Psiquiatría. 2010; 39(4): 771-781
42. Pinilla Analida, Cano Nairo, Granados Carlos, Páez Canro Carol, Eslava-Schmalbach Javier. Inequalities in prescription of hydrochlorothiazide for diabetic hyperthensive patients in Colombia. Revista Salud. Pública, 2011. 13(1): 27-40
43. Eslava-Schmalbach Javier, Rincón Carlos Javier, Guarnizo-Herreño Carol C. [Inequity of Lost Life Years by Departaments in Colombia 1985-2005]. Revista Salud Pública. 2011, 13(1): 1-12
44. Eslava-Schmalbach Javier, Buitrago Gutiérrez Giancarlo, Rincón Carlos Javier. Inequidad de la mortalidad evitable: Conceptos, desarrollos y medición. Bogotá, 2013, Universidad Nacional de Colombia. ISBN: 978-958-761-450-3 y 978-958-761-451-0
45. Eslava-Schmalbach, Javier, Sandoval-Vargas, Gisella, Mosquera, Paola. Incorporating equity into developing and implementing guidelines for evidence-based clinical practice. Revista Salud Pública, 2011; 13 (2): 339-351
46. José Ricardo V. Navarro, Javier Eslava-Schmalbach, Daniel P. R. Estupiñán and Luis A. Carlos Leal (2012). Neurological Complications of Regional Anesthesia, Cesarean Delivery, Dr. Raed Salim (Ed.), ISBN: 978-953-51-0638-8, InTech, Available from: http://www.intechopen.com/books/cesarean-delivery/neurological-complications-of-anesthesia-for-cesarean-section. Accesed: June, 2012. DOI: 10.5772/30765
47. Gomez-Duarte OG, Romero-Herazo YC, Paez-Canro CZ, Eslava-Schmalbach JH, Arzuza O. Enterotoxigenic Escherichia coli associated with childhood diarrhoea in Colombia, South America. J Infect Dev Ctries. 2013;7(5):372-81.
48. Alzate-Granados Juan P, Sánchez-Bello Nubia F, Amaya-Arias Ana C, Peralta-Pizza Fernando, Eslava-Schmalbach Javier. Disparidades en la incidencia de sífilis congénita en Colombia 2005 a 2011: Un estudio ecológico. Rev. salud pública. 2012.14 (6): 71-80. ISSN 0124-0064.
49. Aponte-González Johanna, Rincón Carlos, Eslava-Schmalbach Javier. The impact of under-recording on cervical cancer-related mortality rates in Colombia: an equity analysis involving comparison by provenance. Rev. salud pública. 2012. 14 (6): 912-922
50. Eslava-Schmalbach JH, Rincón CJ, Guarnizo CC. “Inequidad” de la expectativa de vida al nacer por sexo y “departamentos” de Colombia. Biomédica. 2013 Oct 4;33(3):383–92.
51. Aponte-González J, Fajardo-Bernal L, Diaz J, Eslava-Schmalbach J, Gamboa O, Hay JW. Cost-Effectiveness Analysis of the Bivalent and Quadrivalent Human Papillomavirus Vaccines from a Societal Perspective in Colombia. PLoS ONE. 2013 Nov 18;8(11):e80639.
52. Amaya Ana Carolina, Narváez Ricardo, Eslava-Schmalbach Javier. Trabajo en equipo como factor contribuyente en la ocurrencia de errores médicos o eventos adversos. rev. colomb. cir. 2013; 28(4): 297-310
53. Sandoval Vargas, G., Eslava-Schmalbach, J. Inequality regarding maternal mortality in Colombian departments in 2000-2001, 2005-2006 and 2008-2009. Revista de Salud Pública. 2013 Jul;15(4):579–91.
54. Amaya Arias AC, Barajas R, Hernando Eslava Schmalbach J, Wheelock A, Gaitán Duarte H, Hull L, et al. Translation, cultural adaptation and content re-validation of the observational teamwork assessment for surgery tool. Int J Surg 2014; 12 (12): 1390-1402.
55. **Possible evaluators**

Oscar Guevara Cruz MD MSc. Coordinator of the Master's Degree in Clinical Epidemiology, Associate Professor, Department of Surgery. National University of Colombia. E-mail: [oaguevarac@unal.edu.co](mailto:oaguevarac@unal.edu.co)

Hernando Gaitán Duarte MD MSc. Professor, Department of Obstetrics and Gynecology, and the Clinical Research Institute. National University of Colombia. E-mail: [hggaitand@unal.edu.co](mailto:hggaitand@unal.edu.co)

Carlos Gómez MD MSc. Department Director. Associate Professor, Department of Clinical Epidemiology and Biostatistics. Pontifical Javeriana University. E-mail: [cgomez@javeriana.edu.co](mailto:cgomez@javeriana.edu.co)

Ravi Mahajan MD PhD. Professor of Anaesthesia & Intensive Care; Head of Division, Faculty of Medicine & Health Sciences. University of Nottingham. E-mail: [ravi.mahajan@nottingham.ac.uk](mailto:ravi.mahajan@nottingham.ac.uk)

1. **Schedule**

| **No** | **Objective** | **Activity** | **Start month** | **End month** |
| --- | --- | --- | --- | --- |
| 1 | Objectives 1, 2, 3 and 4 | Document organization | 1 | 1 |
| 2 | Objectives 1, 2, 3 and 4 | Contracting personnel | 1 | 1 |
| 3 | Objective 1 | Phase 1. Evidence search, filtering and selection of studies for the systematic literature review of the effectiveness of strategies for knowledge transfer. | 2 | 2 |
| 4 | Objective 1 | Phase 1: Evaluation of the risk of bias in the selected studies, evaluation of the quality of the group of evidence. | 3 | 3 |
| 5 | Objective 1 | Phase 1: Extraction of data and synthesis of the evidence, statistical analysis and production of the final document. | 4 | 4 |
| 6 | Objective 2 | Phase 2: Preliminary design of the content of the health capsules. | 5 | 6 |
| 7 | Objective 2 | Phase 3: Design of the questionnaire and content for formal consensus by experts. | 6 | 7 |
| 8 | Objective 2 | Phase 3: Gathering of experts for formal consensus. | 7 | 7 |
| 9 | Objective 2 | Phase 3: Formal consensus of experts following the modified Delphi method (3 rounds). | 8 | 9 |
| 10 | Objective 2 | Phase 4: Design and production of the knowledge capsules for maternal and neonatal health. | 10 | 11 |
| 11 | Objective 3 | Phase 5: Selection of voluntary participants for neuromarketing strategy. | 11 | 11 |
| 12 | Objective 3 | Phase 5: Application of neuromarketing strategy. | 11 | 13 |
| 13 | Objective 3 | Phase 5: Analysis of results of neuromarketing strategy and final report. | 13 | 13 |
| 14 | Objective 4 | Phase 6: Final production of health capsules based on recommended alterations. | 14 | 14 |
| 15 | Objectives 1, 2 and 3 | Creation of products. | 4 | 14 |
| 16 | Objectives 1, 2 and 3 | Presentation of publications of scientific evidence to editorial committees. | 14 | 16 |
| 17 | Objectives 1, 2 and 3 | National and international presentations. | 14 | 16 |
| 18 | Objectives 1, 2 and 3 | Writing of final report. | 17 | 17 |
| 19 | Objectives 1, 2 and 3 | Close (settlement and act of closure of project). | 18 | 18 |

1. **Possible risks and difficulties**

− Among the possible difficulties that may arise during the execution of the project may be that within the formal consensus of experts there are very diverse points of view that do not allow an agreement level of 80% to be reached, a situation in which a further consultation round will be required to reach the expected level of consensus.

− During the call and selection phase of the sample for the application of the neuromarketing strategy, there may be a problem that women will not agree to participate in the project and there will be limitations in reaching the initially defined sample size.

1. **Environmental impact**

It is expected for a short term, that the implementation of this knowledge transfer strategy will contribute to the knowledge of relevant and clear information for pregnant women and their families and to the empowerment of patients in maternal and neonatal health care. Likewise, it is expected that the results of this research will provide a tool for health professionals that facilitates the use of scientific evidence in clinical decision making in their professional practice for the benefit of the health of the population. In the medium and long term, through the implementation of knowledge capsules in maternal and neonatal health for the preparation of childbirth, disseminated through mass media, it is expected to contribute in the improvement of the health and safety of these patients, with a lower proportion of obstetric complications related to childbirth.

**Bibliography**

1. Rycroft-Malone. Theory and Knowledge TranslationSetting Some Coordinates. Nursing Research. 2007;56(4S):S78-S85.

2. Nora Jacobson DB, Paula Goering,. Development of a framework for knowledge translation: understanding user context. 2003.

3. Secretaría General de las Naciones Unidas. Estrategia Mundial de Salud de las Mujeres y los Niños; Nueva York. Septiembre 2010.

4. Gaitán H. El Instituto Materno Infantil y los planes de reducción de la mortalidad materna: un reto para el sistema de Seguridad Social en Salud. Revista Colombiana de Obstetricia y Ginecología. 2005;56(2).

5. Campbell O, Wendy G. Strategies for reducing maternal mortality: getting on with

what works. Lancet 2006;368:1284–99.

6. Ronsmans C, Graham WJ. Maternal mortality: who, when, where, and why. Lancet 2006;368:1189–200.

7. Panchal S, Arria AM, Labhsetwar SA. Maternal Mortality During Hospital Admission for Delivery: A Retrospective Analysis Using a State-Maintained Database. Anesth Analg 2001;93(134-141).

8. Haynes AB, Weiser TG, Berry WR, Lipsitz SR, Breizat A-HS. A Surgical Safety Checklist to Reduce Morbidity and Mortality in a Global Population. N Engl J Med 2009;360:491-9.

9. Berkman ND, Sheridan SL, Donahue KE, David J. Halpern. Agency for Healthcare Research and Quality. Health Literacy Interventions and Outcomes: An Updated Systematic Review. Evidence Report/Technology Assessment Evidence Number 199. March 2011.

10. Shannon D Scott1* LA, Kathy O’Leary1, Geoff DC Ball2, Lisa Hartling2,3, Anne Hofmeyer4, C Allyson Jones5, Terry P Klassen6,7, Katharina Kovacs Burns8,9, Amanda S N Newton2,10,11, David Thompson and Donna M Dryden,. Systematic review of knowledge translation strategies in the allied health professions. Implementation Science. 2012;7:70.

11. Onil K. Bhattacharyyaa, Elizabeth A. Esteya, Merrick Zwarenstei,. Methodologies to evaluate the effectiveness of knowledge translation interventions: a primer for researchers and health care managers. Journal of Clinical Epidemiology. 2011;64:32-40.

12. Wilson EA, Makoul G, Bojarski EA, Bailey SC, Waite KR, Rapp DN, et al. Comparative analysis of print and multimedia health materials: a review of the literature. Patient Educ Couns. 2012;89(1):7-14.

13. Ministerio de Salud y Protección Social. Análisis de Situación de Salud. Colombia. 2013.

14. World Health Organization: Bridging the ‘know-do’ gap. Geneva: Meeting on knowledge translation in global health; 2006.

15. Say L, Chou D, Gemmill A, Tunçalp Ö, Moller A-B, Daniels J, et al. Global causes of maternal death: a WHO systematic analysis. The Lancet Global Health. 2014;2(6):e323-e33.

16. Kassebaum NJ, Bertozzi-Villa A, Coggeshall MS, Shackelford KA, Steiner C, Heuton KR, et al. Global, regional, and national levels and causes of maternal mortality during 1990–2013: a systematic analysis for the Global Burden of Disease Study 2013. Lancet 2014; 384: 980–1004. 2014;384:980-1004.

17. Ministerio De La Protección Social. Gestión de la Calidad de la Atención en la Salud basada en hechos y datos. Observatorio de calidad de la atención en Salud. Boletines 2,3 y 4. Colombia [cited Junio 2015]. Available from: <http://es.calameo.com/read/000148248c88d41cd384b>.

18. Organización Mundial de la Salud. Lista OMS de verificación de la seguridad de la cirugía, 1ª edición. 2008.

19. Van den Broek NR, Graham WJ. Quality of care for maternal and newborn health: the neglected agenda. BJOG. 2009;116 Suppl 1:18-21.

20. Patabendige M, Senanayake H. Implementation of the WHO safe childbirth checklist program at a tertiary care setting in Sri Lanka: a developing country experience. BMC pregnancy and childbirth. 2015;15:12.

21. Spector JM, Agrawal P, Kodkany B, Lipsitz S, Lashoher A, Dziekan G, et al. Improving quality of care for maternal and newborn health: prospective pilot study of the WHO safe childbirth checklist program. PLoS One. 2012;7(5):e35151.

22. Sebastian V. Neuromarketing and Evaluation of Cognitive and Emotional Responses of Consumers to Marketing Stimuli. Procedia - Social and Behavioral Sciences. 2014;127:753-7.

23. Angela Oatridge AH, Nadeem Saeed, Joseph V. Hajnal, Basant K. Puri, Luca Fusi, Graeme M. Bydder,. Change in Brain Size during and after Pregnancy: Study in Healthy Women and Women with Preeclampsia. AJNR Am J Neuroradiol. 2002;23:19-26.

24. Gabbay J, A. Le May, H. Jefferson, D. Webb, R. Lovelock, J. Powell, and J. Lathlean. (2003). A case study of knowledge management in multi-agency consumerinformed 'communities of practice': implications for evidence-based policy development in health and social services’, Health, 7(3), pp. 283-310,.

25. Valdizán JR. Funciones cognitivas y redes neuronales del cerebro social. REV NEUROL 2008;46((Supl 1)):S65-S8.

26. Learning and Memory. Cognitive Neuroscience Sinauer Associates, Inc; 2010.

27. Benedet MJ. Neuropsicología Cognitiva. Aplicaciones a la clínica y a la investigación Ministerio de Trabajo y Asuntos Sociales M, España, editor2002.

28. Kandel ER, Squire LR. Neuroscience: Breaking down Scientific Barriers to the study of Brain and Mind. SCIENCE. 2000;290:1113-20.

29. Ripoll DR. Neurociencia cognitiva: Editorial Medica Panamericana Sa de; 2013.

30. Javor1 A, Koller M, Lee N, Chamberlain L, Ransmayr aG. Neuromarketing and consumer neuroscience contributions to neurology. BMC Neurology 2013;13(13):1-12.

31. Redolar D. Neurociencia: la génesis de un concepto desde un punto de vista multidisciplinar. Rev Psiquiatría Fac Med Barna. 2002;29(6):346-52.

32. Roberto Álvarez del Blanco. Neuromarketing. Fusión Perfecta. Pearson Educación S.A. Madrid, España. 2011.

33. Ohme R, Reykowska D, Wiener D, Choromanska A. Analysis of neurophysiological reactions to advertising stimuli by means of EEG and galvanic skin response measures. Journal of Neuroscience, Psychology, and Economics. 2009;2(1):21-31.

34. Mele ML, Federici S. Gaze and eye-tracking solutions for psychological research. Cognitive processing. 2012;13 Suppl 1:S261-5.

35. Vecchiato G, Astolfi L, De Vico Fallani F, Cincotti F, Mattia D, Salinari S, et al. Changes in brain activity during the observation of TV commercials by using EEG, GSR and HR measurements. Brain topography. 2010;23(2):165-79.

36. Zamora MEC, Rubilar FC, Ramos HL. Estudio descriptivo de las estrategias cognitivas y metacognitivas de los alumnos y alumnas de primer año de pedagogía en enseñanza media de la Universidad del Bío-Bío Theoria. 2004;13:103-10.

37. Chevalier TM, Parrila R, Ritchie KC, Deacon SH. The Role of Metacognitive Reading Strategies, Metacognitive Study and Learning Strategies, and Behavioral Study and Learning Strategies in Predicting Academic Success in Students With and Without a History of Reading Difficulties. Journal of learning disabilities. 2015.

38. Sevilla JG. Estimulación Cognitiva. Estimulación en Estrategias Cognitivas y Metacognitivas: Martín Antonio Vega Tineo; 2014.

39. Wilson EA, Wolf MS. Working memory and the design of health materials: a cognitive factors perspective. Patient education and counseling. 2009;74(3):318-22.

40. Graham ID LJ, Harrison MB, Straus SE, Tetroe J, Caswell W, Robinson N,. Lost in knowledge translation: time for a map? J Contin Educ Health Prof. 2006;26(1):13-24.

41. Davis RE, Jacklin R, Sevdalis N, Vincent CA. Patient involvement in patient safety: what factors influence patient participation and engagement? Health expectations : an international journal of public participation in health care and health policy. 2007;10(3):259-67.

42. Organización Mundial de la Salud. Trabajando con Individuos, familias y comunidades para mejorar la salud materna y neonatal. 2010.

43. Okoli C, Pawlowski SD. The Delphi method as a research tool: an example, design considerations and applications. Information & Management. 2004;42(1):15-29.

44. Balasubramanian R, Agarwal D. Delphi Technique- A Review. International Journal of Public Health Dentistry. 2012;3(2):16-25.

45. Hsu C-C, Sandford BA. The Delphi Technique: Making Sense Of Consensus. Practical Assessment, Research & Evaluation. 2007;12(10).

46. DECLARACION DE HELSINKI DE LA ASOCIACION MEDICA MUNDIAL. Principios éticos para las investigaciones médicas en seres humanos DoH Oct 2008 [cited junio 2011]. Available from: <http://www.wma.net/es/30publications/10policies/b3/17c_es.pdf>.

47. REPUBLICA DE COLOMBIA. MINISTERIO DE SALUD. RESOLUCION Nº 008430 DE 1993.

1. **Environmental permits and licenses**

The present project does not require any environmental permit or license for its execution.

**Appendix 1**

**CONSENT TO PARTICIPATE IN A PILOT STUDY FOR THE EVALUATION OF EFFECTIVENESS OF KNOWLEDGE CAPSULES IN MATERNAL AND NEONATAL HEALTH THROUGH NEUROMARKETING STRATEGIES**

HEALTH EQUITY GROUP

GROUP OF QUALITY, SECURITY AND HEALTH EDUCATION

NEUROSCIENCE GROUP

National University of Colombia

Colombian Society of Anesthesiology and Resuscitation

**Principal Investigator**: Javier Hernando Eslava

**Co-Investigators**: Ana Carolina Amaya, Maria Fernanda Lara

Dear Participant:

You have been invited to participate in the project "PILOT STUDY FOR THE EVALUATION OF EFFECTIVENESS OF CAPACITIES OF KNOWLEDGE IN MATERNAL AND NEONATAL HEALTH THROUGH NEUROMARKETING STRATEGIES"

We will explain to you what the study consists of:

*If this consent contains some words that you do not understand, please ask for an explanation from one of the members of the research group for advice.*

*Before making the decision to participate in the research, carefully read this consent form and discuss with the researcher any concerns you may have.*

Research studies are designed to improve scientific knowledge that may be useful to other people in the future. You may not receive any direct benefit for your participation. Your participation is voluntary, you may refuse to participate, or you may withdraw your consent at any time and for any reason.

It is important that you understand the following information to be able to decide in a free and informed manner if you wish to participate in this investigation.

**Purpose of this study**

In this project we propose to design and evaluate the effectiveness of 12 knowledge capsules in maternal and neonatal health to generate appropriation of knowledge in the target population. That is, to design 12 short videos that contain important information to improve the health of pregnant women, their safety during and after childbirth and the safety of the newborn.

This is in order to improve the education and information that this population has about the birth process, either natural or by cesarean section, and the care and behaviors that a patient must assume to contribute along with the health team to their safety.

**Process**

During the investigation the following steps will be carried out:

Short videos, approximately 40 seconds long, will be screened, each one containing topics related to the preparation for childbirth and postpartum.

During the projection, some measurements will be made, which include the use of two electrodes to measure the galvanic response of the skin. This means that two sensors will be placed on two of your fingers that will measure your perspiration, palpitation and electrical response. In addition, you will be sitting in front of a computer that has an infrared meter of your pupillary response, that is, the response your eyes have to the images you see.

It is important to clarify that none of these devices is invasive: the battery will not be punctured, and no electricity or any type of wave will happen that could cause harm to you or your baby in the event that you are pregnant.

Afterwards, there will be a survey in which some questions will be asked related to what you observed in the videos.

**Disadvantages and risks**

The measurements that will be carried out and the application of surveys are considered procedures without medical risk.

**Confidentiality**

All information obtained and the results of the investigation will be treated confidentially. This information will be filed in paper and electronic media. The study file will be under the responsibility of the researchers.

I certify that I have read and understood the information previously provided. I voluntarily agree to participate in this study.

Signature: _________________________________

Identity card: ____ No. ______________ of ____________

Date:__________________
