# Supplementary material for: Effectiveness assessment of maternal and neonatal health video clips in knowledge transfer using neuromarketing tools: A randomized crossover trial
Source: PLoS One. 2019 May 8;14(5):e0215561. doi: 10.1371/journal.pone.0215561 (PMC6505891; doi:10.1371/journal.pone.0215561)
Supplement: S2 File — (DOCX) [file pone.0215561.s003.docx]

**S2 File. Phases of the evaluation study for the effectiveness of 12 video clips of knowledge transfer in maternal and neonatal health using applied neuroscience tools: a randomized crossover clinical trial**

This study was carried out in 6 phases:

Phase 1: Two systematic reviews carried out to evaluate: 1) the effectiveness of the strategies of knowledge transfer in audiovisual language compared with others to improve outcomes in the health of the individual and in the general population (previously published study [1]), and 2) the effectiveness of different audiovisual strategies used in the transfer and acceptance in society of health knowledge. The main conclusions were:

• For a KT strategy, the use of redundant elements should be avoided in the design of animated audiovisual formats; however, text on the screen, presented as action-oriented keywords, can help in the retention of information.
• The presence or absence of a pedagogical agent on screen does not have any additional positive effect, and the narration of the material, presented in a human voice with a standard accent and with elements of personalized narration (in which the word "he" is replaced by the word "you"), improves both the recall and the understanding of the information.
• 3D formats are a tool that provides a better recall of information compared to 2D formats and narrative formats, and provide advantages over traditional information-based formats, especially in interventions aimed at reducing health inequalities.

Phase 2: Preliminary definition of the content of the video clips of awareness in maternal and neonatal health. The design of the 12 video clips was created by the researchers of the group, and the proposal was presented to a group of external experts in Phase 3.

Phase 3: Formal consensus of experts using the modified Delphi method to define the content and strategies to be used in them. Ten experts in gynecology and obstetrics, psychology and health education, promotion and prevention programs were convened, each with at least 5 years of experience in maternal and perinatal health (Table 1).

Table 1. Experts and participants in Phases 2 and 3.

| Name | Area | Function in the panel of experts |
| --- | --- | --- |
| Diana Arias | Neonatology | Technical details in the subject of care of the newborn child |
| Dario Sotelo | Gynecology and Obstetrics | Technical details in the subject of obstetrics |
| Viviana Andrea Camacho | Gynecology and Obstetrics | Technical details in the subject of obstetrics |
| Olga Lucía Vaquero | Pediatrics | Technical details in the subject of pediatrics |
| Claudia Sotomayor | Psychology | Psychosocial support |
| Sandra Carolina Fagua García | Psychology | Psychosocial support |
| Nancy Bejarano | Nursing | Patient education |
| Rosalina Sánchez | Nursing | Patient education |
| Diana Caballero | Nursing | Patient safety |
| Adriana Osorio | Medicine | Proposal and prevention |

Following phases 2 and 3, the contents presented in Table 2 were defined, such as the thematic content, characteristics and areas of interest that should be included in the 12 video clips. These were categorized in three stages: 4 for the prenatal stage, 4 for the stage of childbirth and 4 for the postpartum.

Table 2 Video clips of maternal and neonatal health knowledge

| **Video-clip*** | **Stage** | **Aspects finally included** | **Areas of interest** | **Characteristics** |
| --- | --- | --- | --- | --- |
| Video-clip 1 | Prenatal stage | Healthy eating guidelines in pregnancy. Evaluation before birth. Prenatal consultation (checklist with documents and exams), importance of bringing maternity card to the consultations, number of prenatal consultations. Suspension of the consumption of alcohol and psychoactive substances. Referral for dental evaluation. Importance of communication with the doctor of prenatal care in case of doubts. Correct use of the safety belt during pregnancy. | • Cheese • Meat • Apple • Forbidden substances • Dental evaluation • Seat belt | Duration: approximately 1 minute 18 seconds Characters: - Expectant mother in first trimester. - Doctor. - Nurse. Location: - Maternal care center and prenatal check-up clinic. |
| Video-clip 2 | Prenatal stage | Importance of dietary supplements during pregnancy (iron, folic acid and calcium). Management of symptoms during pregnancy (diet, do not self-medicate or take supplements not prescribed, inform the doctor about symptoms). | • Folic acid, iron, calcium • Doctor • Do not self-medicate • Fruits, vegetables, water • Folic acid, calcium, iron • No supplements | Duration: approximately 1 minute 20 seconds Characters: - Expectant mother in pre-conception consultation. - Doctor. - Expectant mother in third trimester. Locations: - Prenatal check-up clinic. - Home of expectant mother in third trimester. |
| Video-clip 3 | Prenatal stage | Exams during prenatal check-ups, ultrasounds and vaccines during pregnancy. Importance of registering in the prenatal control database. Recommendations to prevent vector-borne diseases in risk areas. | • Blood test and mid-term urine test • Vaccines • Young Adriana in a hammock • Ultrasound • Growing belly • List of exams • Baby | Duration: approximately 1 minute 18 seconds  Characters: - Expectant mother in first trimester (Adriana). - Doctor. Location: Maternal care center. |
| Video-clip 4 | Prenatal stage | Signs of alarm during pregnancy (urinary infection, edema in lower limbs, symptoms of pre-eclampsia, rupture of membranes, perception of movements of the fetus, hemorrhage, coughing, fever, weakness or vertigo). Possible complications during pregnancy and warning signs | • Alarm signs • Belly • Feet • Liquid • Doctor • Burning sensation • Exams  • Emergency signs | Duration: approximately 1 minute 30 seconds  Characters: - Expectant mothers. - Doctor. - Nurse. Locations: - Maternal care center and prenatal check-up clinic. - Hospital emergency care center. |
| Video-clip 5 | Childbirth stage | Information to the pregnant woman about cesarean sections (indications, local anesthesia, procedure, risks, pain management, signing of form of consent). | • Title text (Caesarean section) • Signs in the folder • Medical records • Photo in the medical record (image in the medical record) • Suture with red circle, rays and drop • Doctor • Consent (text of form of consent) • Doctor signature • Patient signature • Check list | Duration: approximately 1 minute 13 seconds  Characters: - Expectant mother in third trimester. - Doctor.   Locations: - Prenatal check-up clinic. - Surgery room for the cesarean section. |
| Video-clip 6 | Childbirth stage | Start of labor, initial assessment and signs of re-consultation in case of not being admitted to the institution (count of contractions for identification of active phase of labor). Humanized childbirth guidelines (company of the partner). | • Title text (Start of labor) • Adriana • Taking blood pressure • Stethoscope • Enlarged clock • Adriana • Nurse | Duration: approximately 1 minute 03 seconds Characters: - Partner - Expectant mother in third trimester - Nurse Locations: - Expectant mother’s home. - Consulting room at childbirth center. |
| Video-clip 7 | Childbirth stage | Admission to the health institution and explanation of labor up to its active phase (evaluations, importance of consultation regarding doubts). | • Title text • Hospital image - hospital text • Pregnant woman touching her abdomen due to contractions • Sequence of increasing points on graph • Text: First childbirth = 8 to 18 hours and subsequent childbirths = 5 to 12 hours • Text: frequency of contractions • Text: blood pressure • Pelvic exam text • Husband embracing pregnant woman | Duration: approximately 1 minute 11 seconds Characters - Nurse - Expectant mother in third trimester - Partner  Location: - Consulting room at childbirth center. |
| Video-clip 8 | Childbirth stage | Explanation types of analgesia during labor Humanized delivery guidelines (accompaniment during labor). | • Title text • Text: analgesia • Woman shaking her head • Needle and rising blue color in arm • Doctor applying medications in saline solution • Pain of the pregnant woman and arms of the partner | Duration: approximately 1 minute 05 seconds Characters: - Doctor - Expectant mother Locations: - Surgery room, delivery room. |
| Video-clip 9 | Postpartum stage | Skin to skin contact of the mother and the newborn. Breastfeeding. Recognition of warning signs for the mother and the newborn | • Title text: Skin-to-skin contact and early breastfeeding • Abdomen and face of the woman • Doctor delivering baby to the mother • Mother's face • Husband of the mother embracing her • Newborn breastfeeding • Tying umbilical cord • Doctor with stethoscope examining newborn • Newborn (face and torso) on the mother | Duration: approximately 55 seconds Characters: - Mother immediately after childbirth. - Newborn child. Location: - Maternity ward. |
| Video-clip 10 | Postpartum stage | Supplementation and neonatal prophylaxis, vaccination, screening activities | • Text title: Adaptation of the newborn child • Cleaning text • Nurse cleaning the newborn • Text: cutting umbilical cord • Cutting cord and drying with gauze • Application of drops in baby's eye • Eye prophylaxis text • Vaccination text • Applying injection to baby’s thigh • Meter • Scale numbers • Text: breastfeeding position • Breastfeeding baby • Doctor delivering order (paper) | Duration: approximately 58 seconds Characters: - Newborn child. - Mother immediately after childbirth. - Nurse. Location: - Place of neonatal adaptation. |
| Video-clip 11 | Postpartum stage | Advice on postpartum family planning methods (barrier, definitive and hormonal). Prevention of sexually transmitted diseases with the use of condoms. Referral for administration | • Family Planning Text • Doctor • Ximena agreeing • Images of methods that come out of the folder • Doctor 2 • Ximena • Text: planning methods • Images of planning methods | Duration: approximately 48 seconds Characters: - Woman after childbirth. - Doctor. Location: - Prenatal check-up clinic. |
| Video-clip 12 | Postpartum stage | Discharge and postpartum recovery (early stages) - Signs of alarm in the mother and the newborn (vaginal delivery and caesarean section) | • Text: Post-partum Recovery • Review of images of the 3 women • Doctor • Parents and baby in crib • Grandmother with mask • Image of faucet • Images of baby lying on its back • Text: lying on its back • Avoid objects in the crib • Mother cleaning her wound • Emergency signs • Mother and baby • Review of images of the 3 women | Duration: approximately 1 minute 05 seconds Characters: - Newborn child. - Expectant mothers. Locations: - House, exteriors. |
| *Technical characteristics of video clips: Animation created with mixed 2D and 3D animation, in color, which includes characters with human voices, in HD quality (1280x720px.) and with stereo sound.  Control video: Duration 42 seconds, in 2D, with information on prenatal control presented in conventional format (narration, static images and text on screen)  Source: Authors | | | | |

Phase 4: Design and development of the 12 video clips of maternal and neonatal health based on expert judgment. The video clips were created using 2D and 3D animation techniques, under the supervision of experts in audiovisual media and based on the results of the previous phases. The production of the videos included the selection and approval of the characters, the environments and the voices, and the writing and approval of the scripts. The researchers defined areas of interest (elements of the video that contained the central message of the clip and therefore must be observed by the majority of the participants) for each segment of the 12 videos. Figure 1 shows a capture of each video clip and the control video.

Figure 1. Video clips on maternal and neonatal health

| **Video-clip 1. Prenatal control** | **Video-clip 2. Symptoms during pregnancy** |
| --- | --- |
| **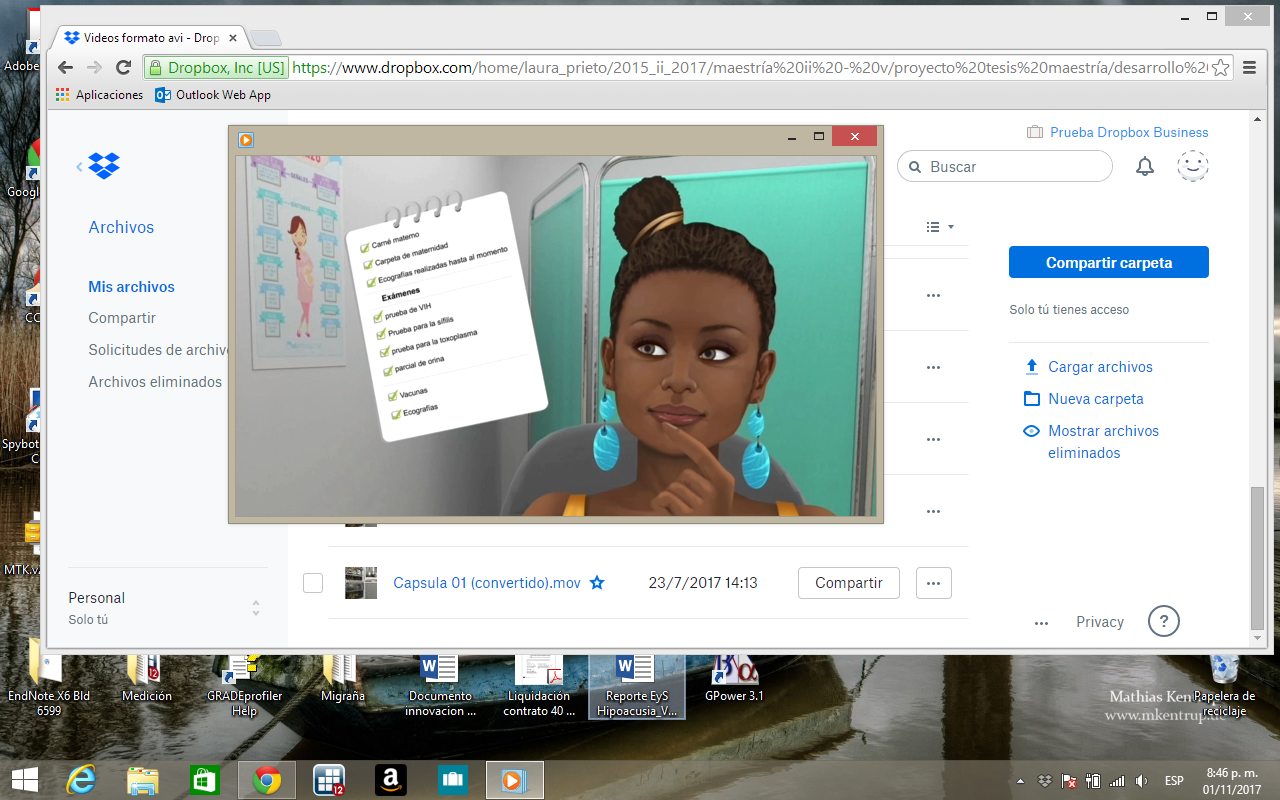** | **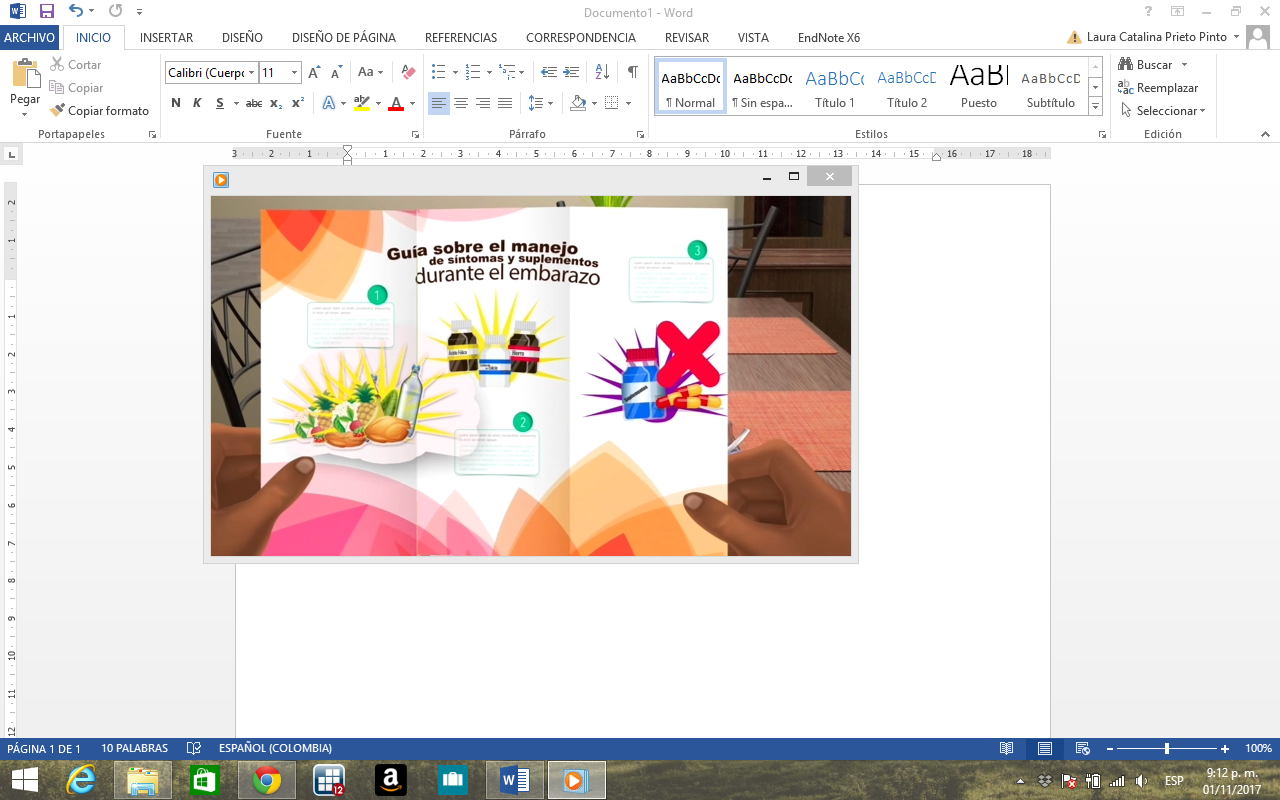** |
| **Video-clip 3. Examinations during pregnancy** | **Video-clip 4. Warning signs during pregnancy** |
| **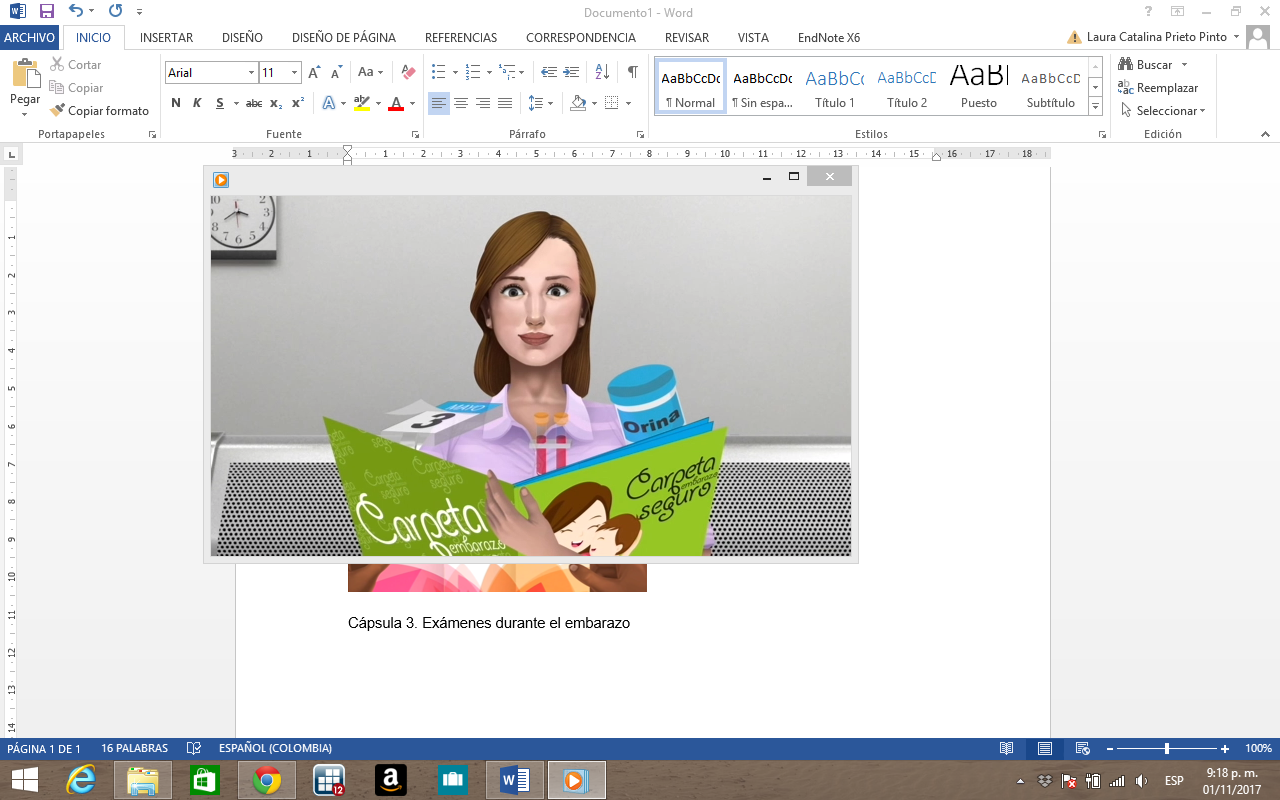** | **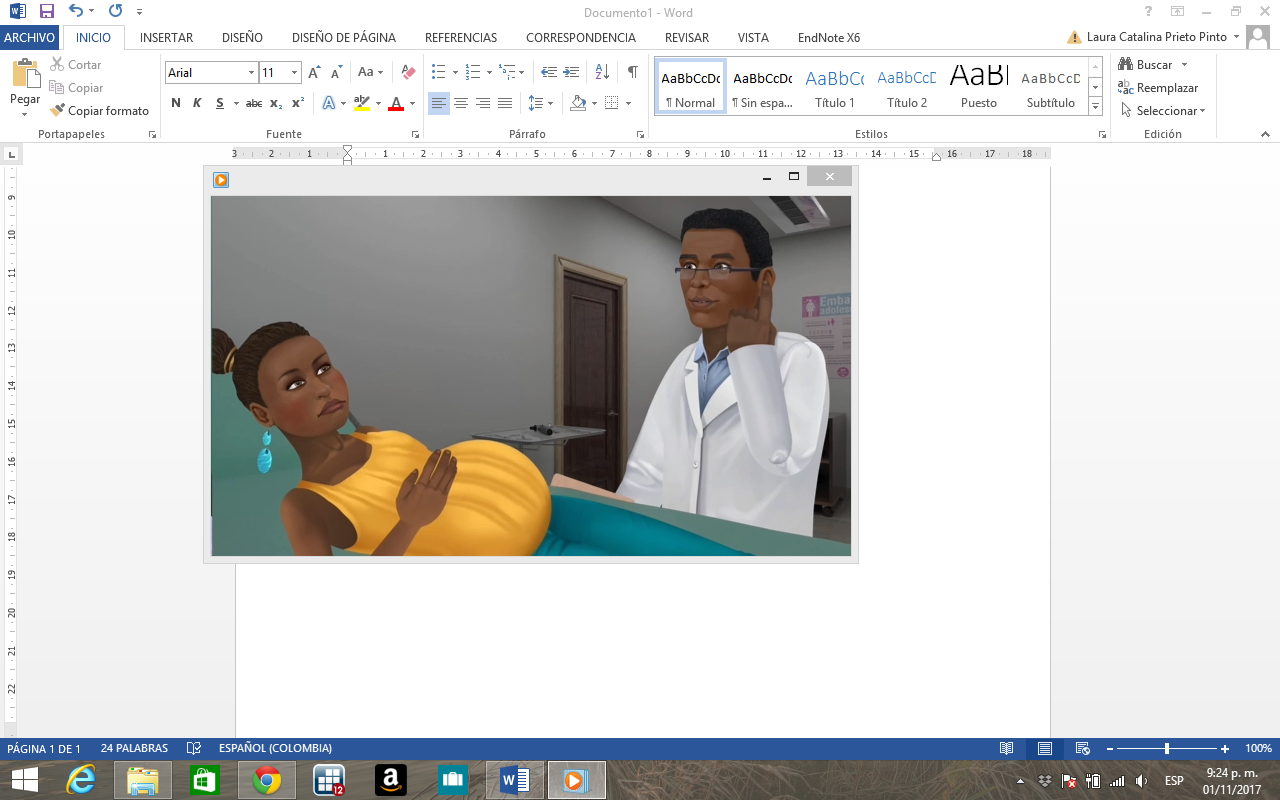** |
| **Video-clip 5. Cesarean sections** | **Video-clip 6. Start of labor** |
| **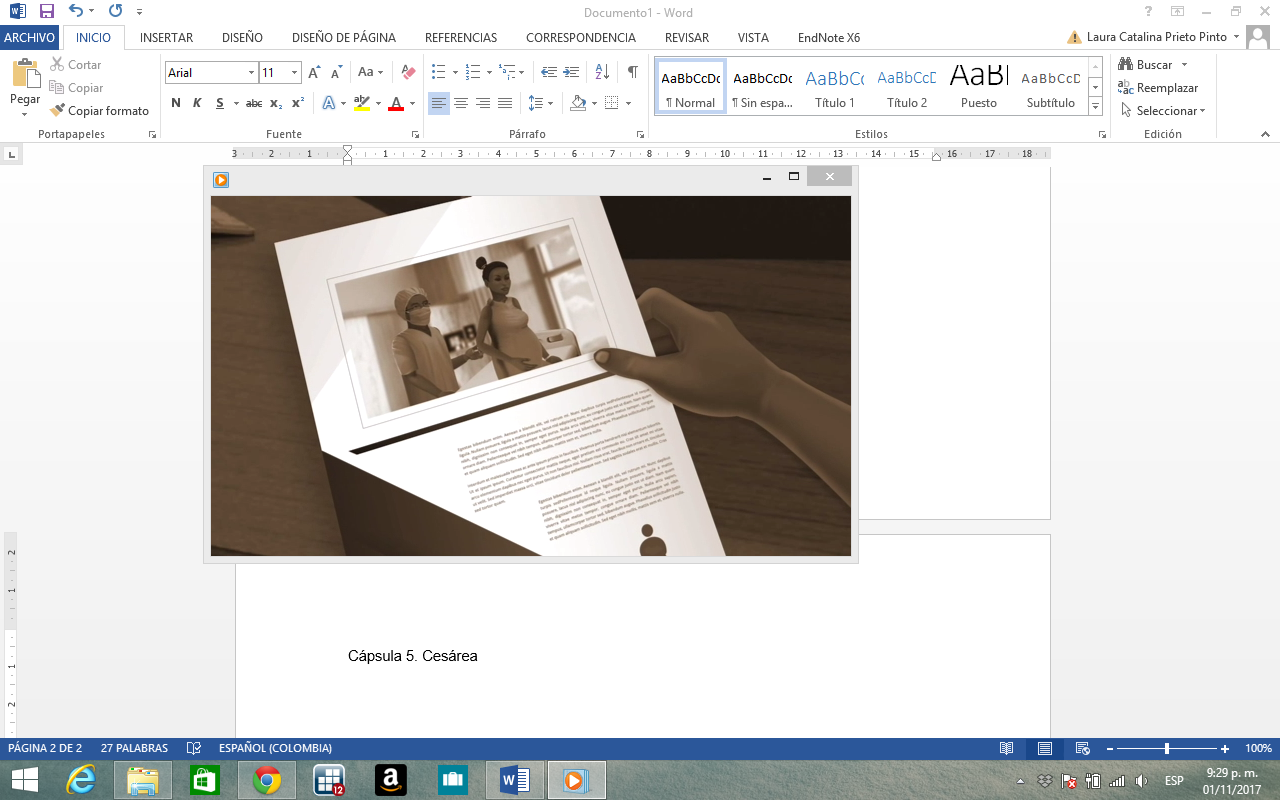** | **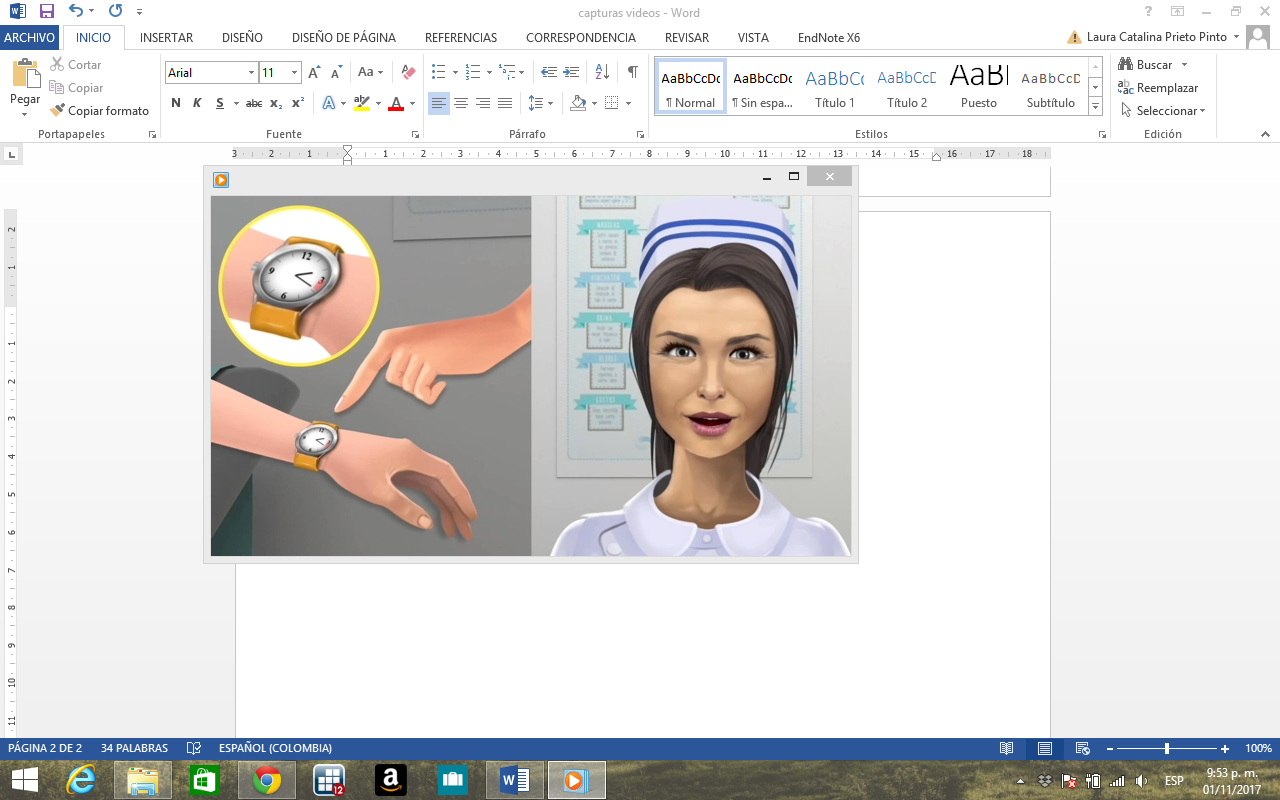** |
| **Video-clip 7. Labor** | **Video-clip 8. Pain management** |
| **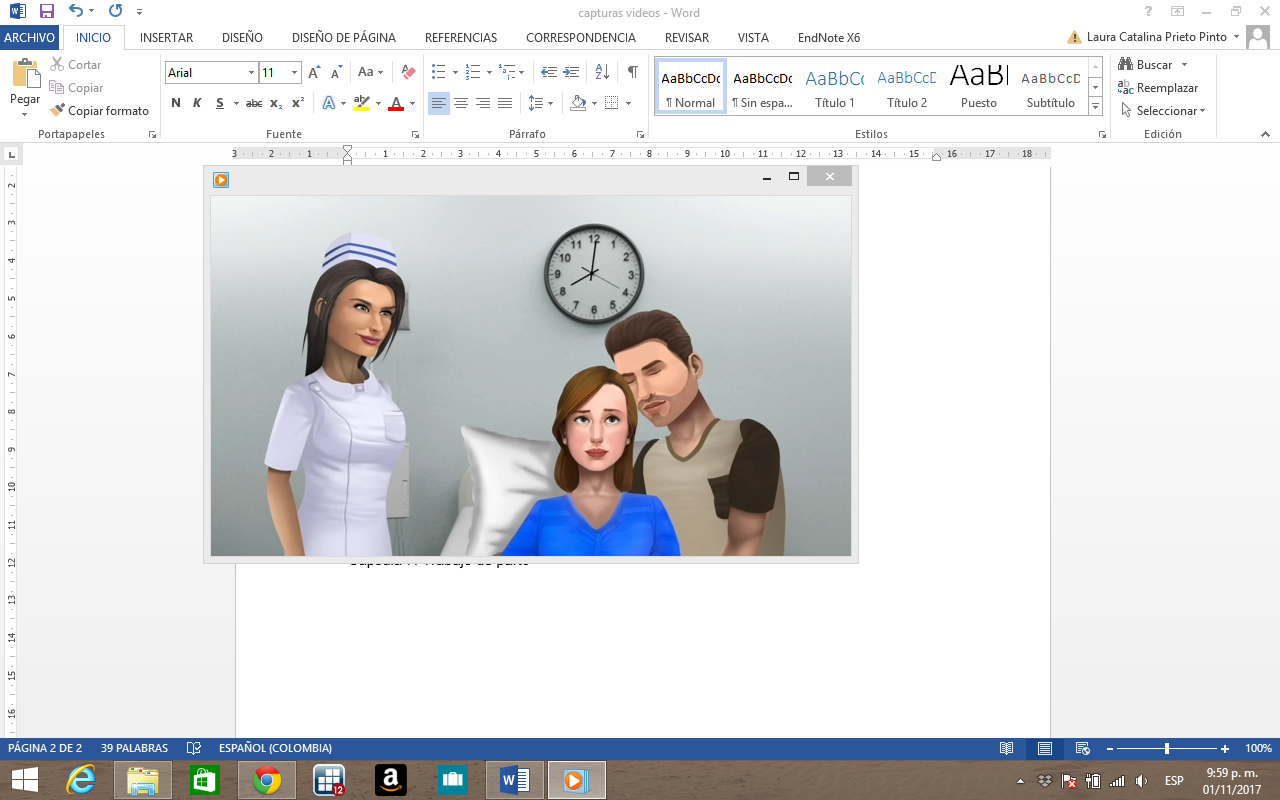** | **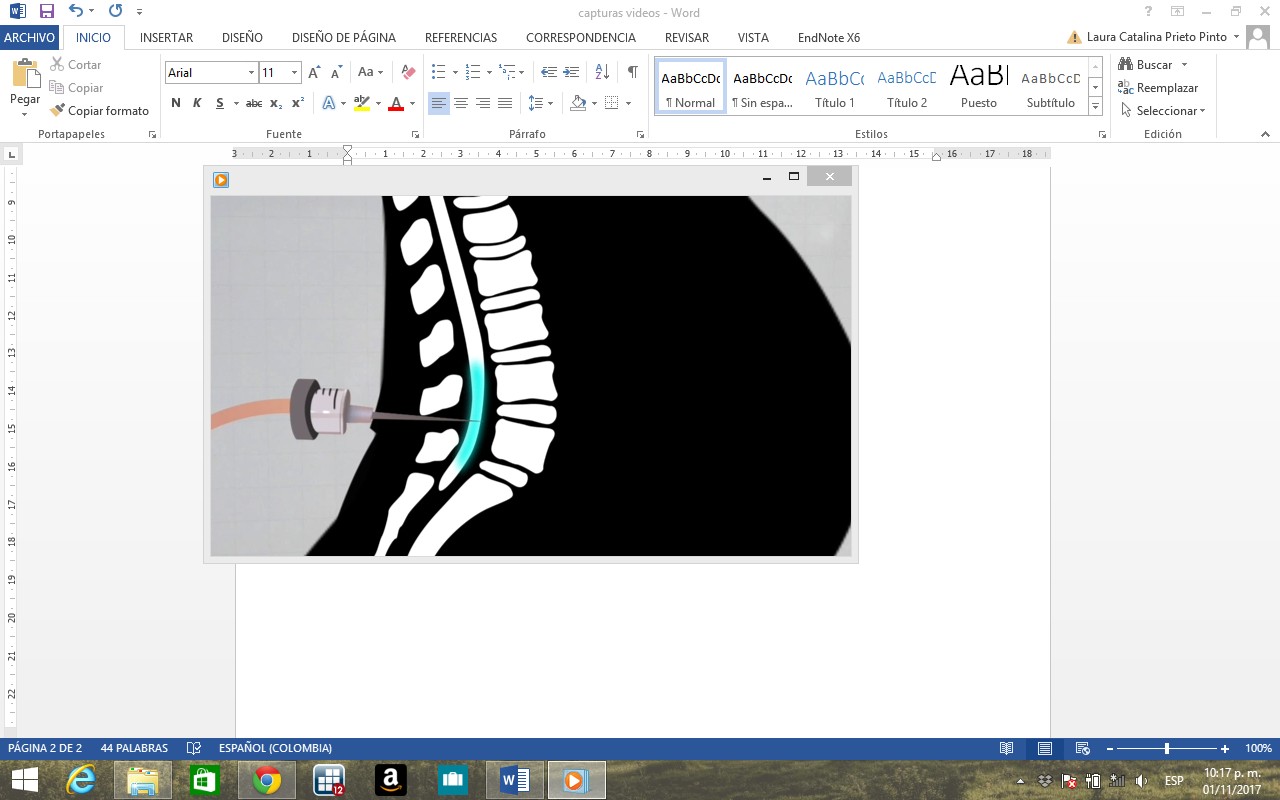** |
| **Video-clip 9. Skin-to-skin contact and early breastfeeding** | **Video-clip 10. Adaptation of newborn child** |
| **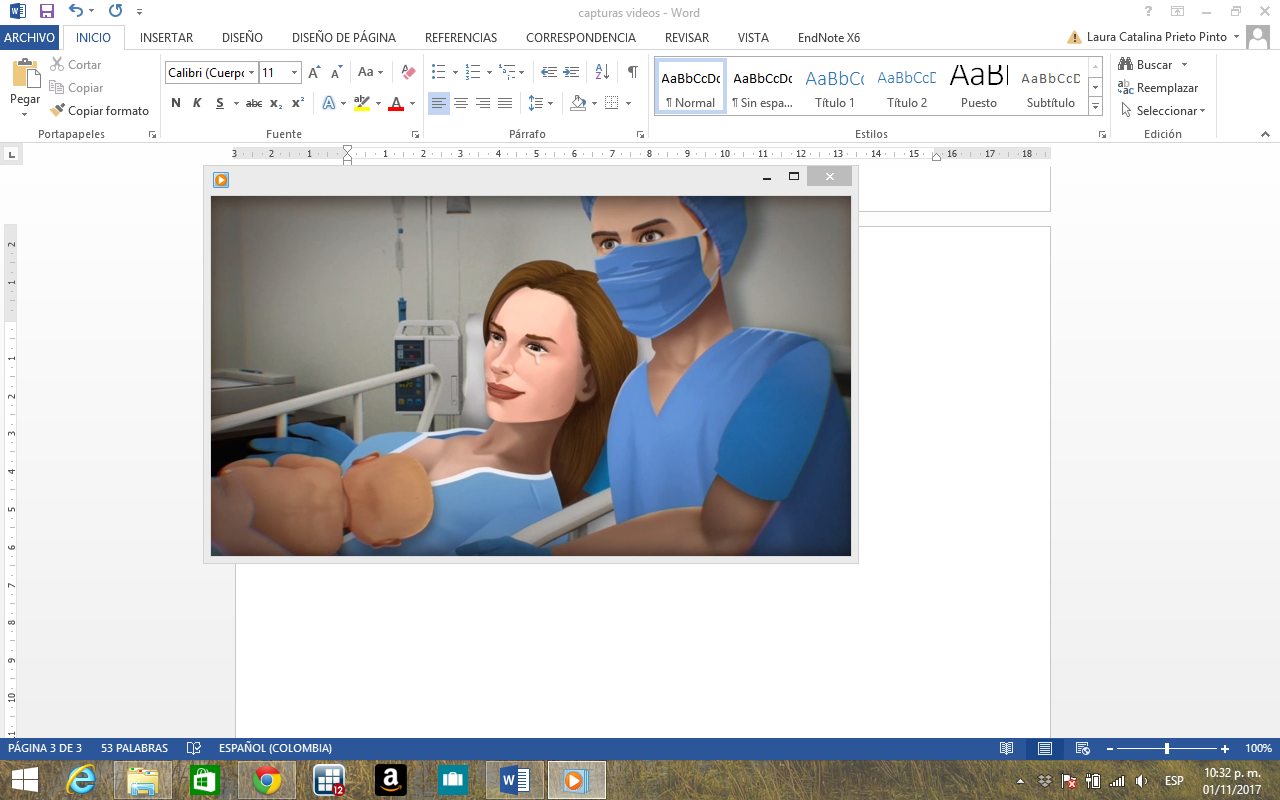** | **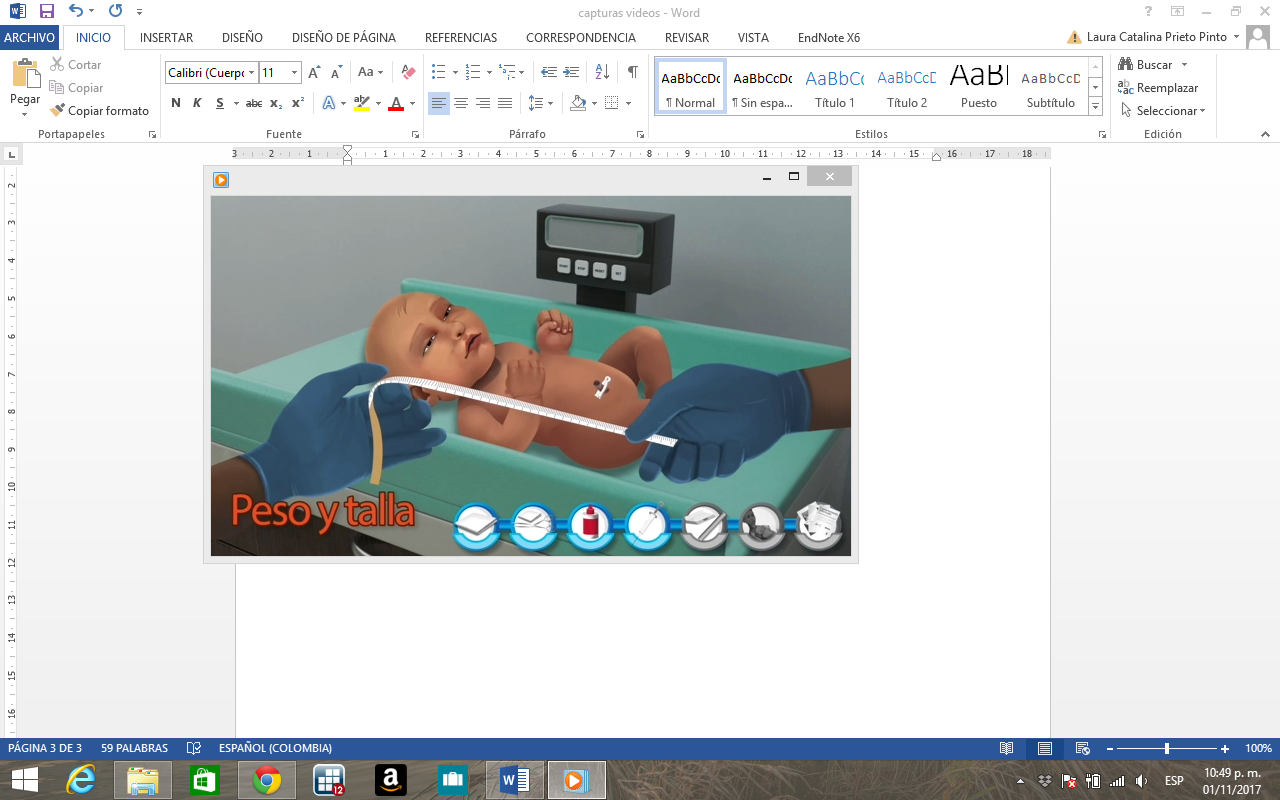** |
| **Video-clip 11. Family planning** | **Video-clip 12. Recovery after birth** |
| **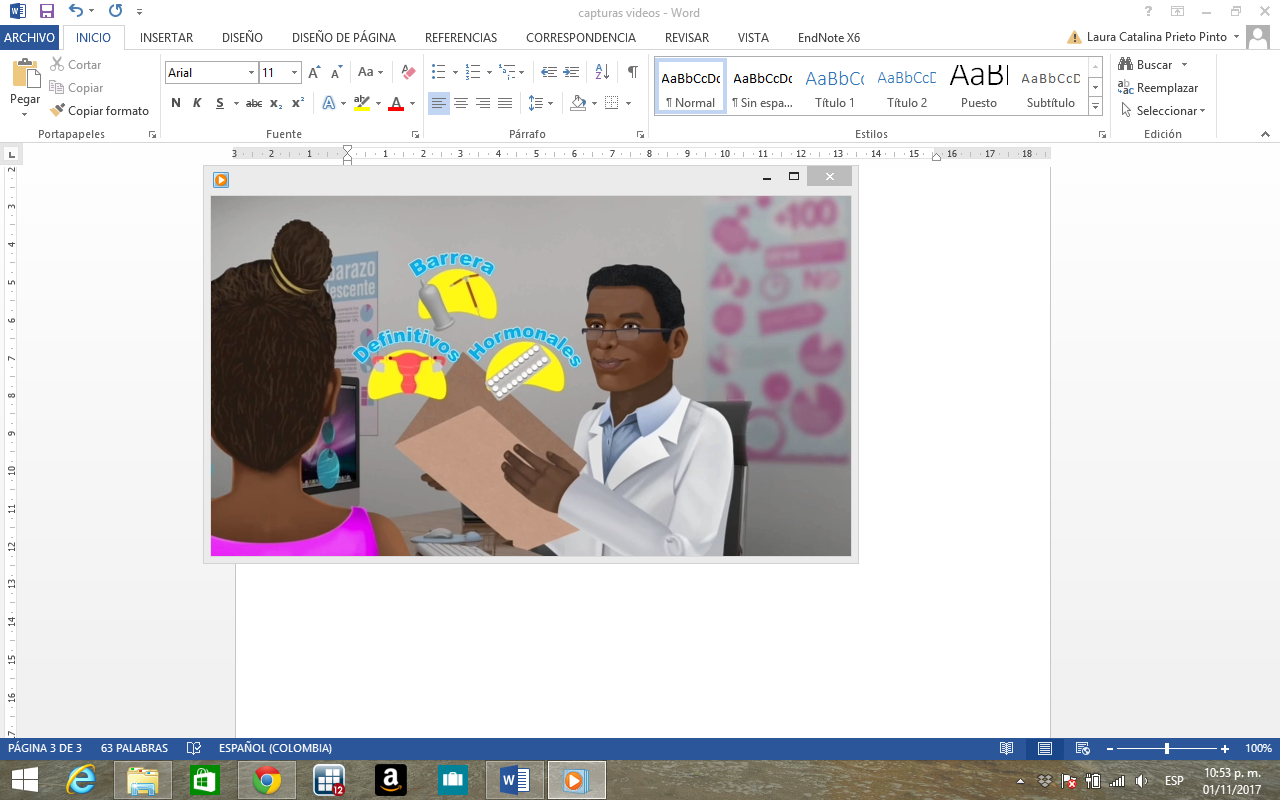** | **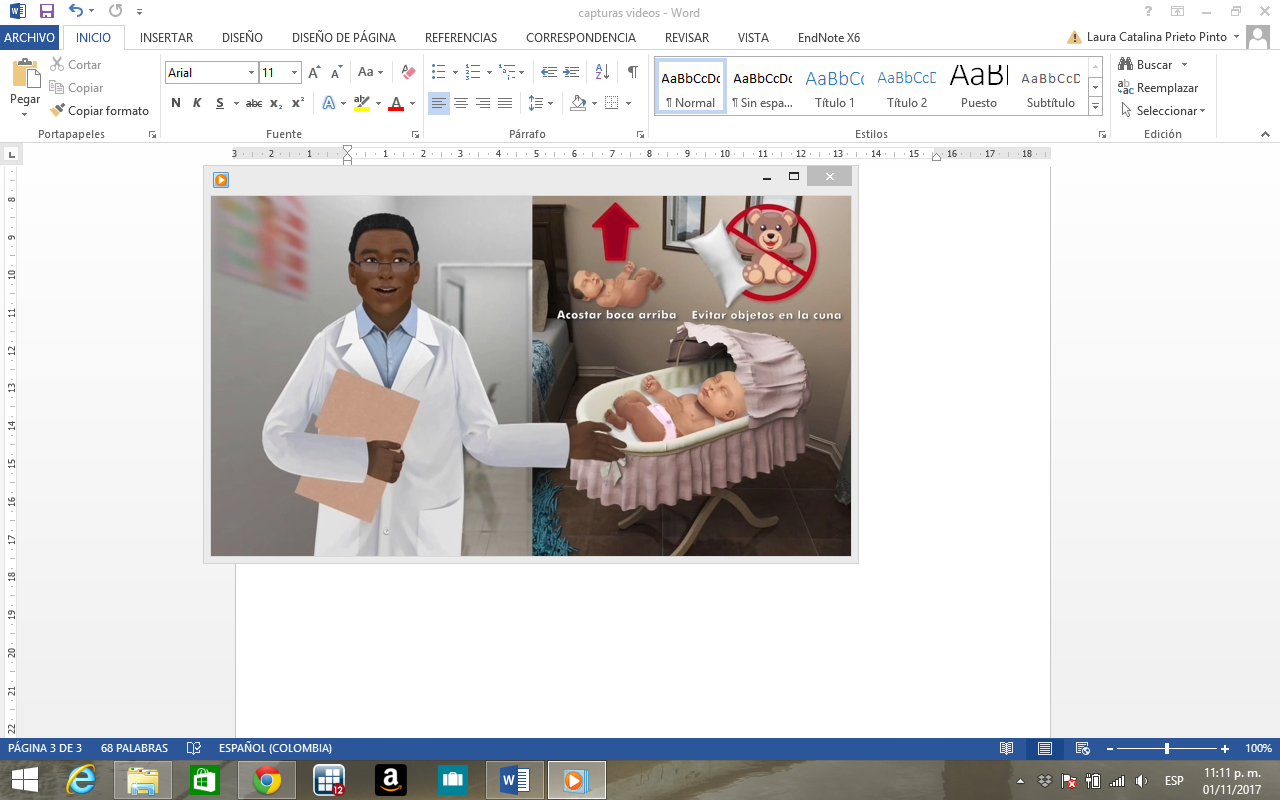** |
|  |  |
| **Video-clip 13. Control video** | |
| **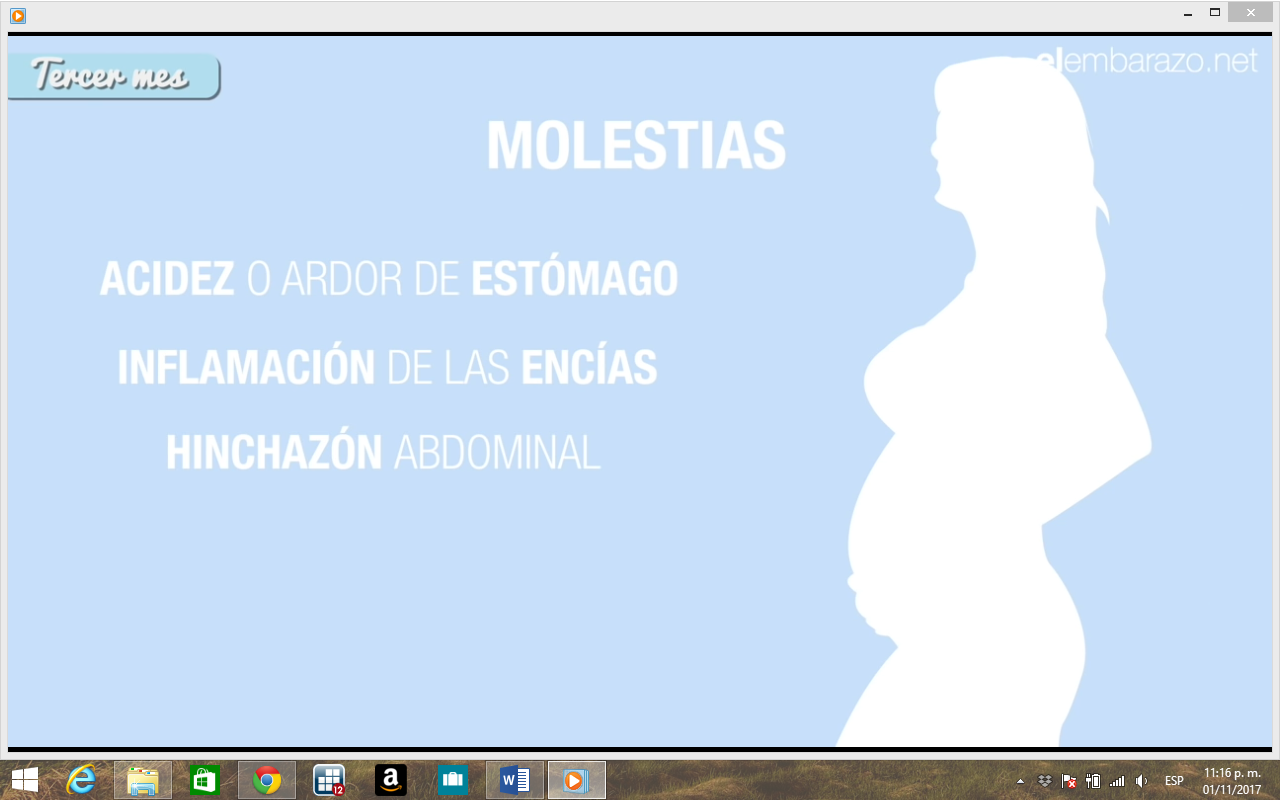** | |

Phase 5: Evaluation of the effectiveness of the video clips in maternal and neonatal health through a neuromarketing strategy via a crossover experimental study (results of the study).

Phase 6: Final production of health knowledge video clips in maternal and neonatal safety. The number of visual fixations was calculated for each area of interest: those that obtained a low percentage of fixations were identified as low attention and editing and design improvements were made.

References:

1. Garzón-Orjuela N, Sánchez Bello NF, Bonilla Mahecha LP, Moreno Hernández LA, Suárez Ángel MC, Murcia Ardila NV, et al. Efectividad de las estrategias de transferencia de conocimiento en lenguaje audiovisual comparadas con otras para mejorar desenlaces en la salud individual y de la población general: revisión sistemática. Rev Colomb Psiquiatr [Internet]. 2017 Sep; Available from: http://linkinghub.elsevier.com/retrieve/pii/S003474501730094X
