# Supplementary material for: Effectiveness assessment of maternal and neonatal health video clips in knowledge transfer using neuromarketing tools: A randomized crossover trial
Source: PLoS One. 2019 May 8;14(5):e0215561. doi: 10.1371/journal.pone.0215561 (PMC6505891; doi:10.1371/journal.pone.0215561)
Supplement: S3 File — (DOCX) [file pone.0215561.s004.docx]

**S3 File. Recall questionnaires of video clips 1 (prenatal control) and 4 (warning signs during pregnancy)**

Video clip 1 (prenatal control)

Bellow you will find some questions related to the video clip 1 (prenatal control). Please select the correct answer.

**1. How should you eat your food?**

A: Raw

B: Unwashed

C: Cooked and with valid expiration date.

D: Expired

**2. What are the documents that you should bring to prenatal control appointment?**

A: All exams, ultrasounds and vaccination certificates

B: You do not have to bring any document

C: Only the pregnancy test

D: Only the pregnancy ultrasounds

**3. How many prenatal control appointments should you attend during pregnancy?**

A: Fifteen prenatal control appointments

B: Only one prenatal control appointment

C: None

D: From 7 to 10 prenatal control appointments

**4. In addition to prenatal control appointments, what other appointments should you attend during pregnancy?**

A: Internal medicine

B: Dentistry and parents training course

C: Gastroenterology

D: None

Video clip 4 (warning signs during pregnancy)

Bellow you will find some questions related to the video clip 4 (warning signs during pregnancy). Please select the correct answer.

**1. What happened to Jimena (mom 2)?**

A: She had swollen ankles, headache and burning in the mouth of the stomach

B: She was in the parents training course

C: Nothing

D: She was in her prenatal control appointment

**2. Some tests will be taken to Jimena to rule out:**

A: Intestinal infection

B: Toxoplasmosis

C: Preeclampsia

D: Appendicitis

**3. ¿What happened to María (mom 3)?**

A: Nothing

B: She had leakage of fluid

C: She went to take some control exams

D: She was at the marketplace

**4. If bleeding occurs during pregnancy, ¿what should be done?**

A: Bed rest

B: Request ambulatory appointment

C: Call a friend

D: Go immediately to the emergency department
